# Cleaved TMEM106B forms amyloid aggregates in central and peripheral nervous systems

**DOI:** 10.1186/s40478-024-01813-z

**Published:** 2024-06-17

**Authors:** Mehtap Bacioglu, Manuel Schweighauser, Derrick Gray, Sofia Lövestam, Taxiarchis Katsinelos, Annelies Quaegebeur, John van Swieten, Zane Jaunmuktane, Stephen W. Davies, Sjors H. W. Scheres, Michel Goedert, Bernardino Ghetti, Maria Grazia Spillantini

**Affiliations:** 1 Department of Clinical Neurosciences, University of Cambridge, Cambridge, UK; 2 Medical Research Council Laboratory of Molecular Biology, Cambridge, UK; 3 IUSM Center for Electron Microscopy (ICEM), Indiana University School of Medicine, Indianapolis, IN USA; 4 Cambridge University Hospitals NHS Foundation Trust and the Cambridge Brain Bank, Cambridge, UK; 5 Department of Neurology, Erasmus Medical Centre, Rotterdam, The Netherlands; 6 Division of Neuropathology, National Hospital for Neurology and Neurosurgery, Queen Square, London, UK; 7 Department of Clinical and Movement Neurosciences, UCL Queen Square Institute of Neurology, London, UK; 8 Department of Cell and Developmental Biology, University College, London, UK; 9 Department of Pathology and Laboratory Medicine, Indiana University School of Medicine, Indianapolis, IN USA

**Keywords:** TMEM106B filaments, Amyloid, Astrocytes, Vacuoles, Peripheral nervous system

## Abstract

Filaments made of residues 120-254 of transmembrane protein 106B (TMEM106B) form in an age-dependent manner and can be extracted from the brains of neurologically normal individuals and those of subjects with a variety of neurodegenerative diseases. TMEM106B filament formation requires cleavage at residue 120 of the 274 amino acid protein; at present, it is not known if residues 255-274 form the fuzzy coat of TMEM106B filaments. Here we show that a second cleavage appears likely, based on staining with an antibody raised against residues 263-274 of TMEM106B. We also show that besides the brain TMEM106B inclusions form in dorsal root ganglia and spinal cord, where they were mostly found in non-neuronal cells. We confirm that in the brain, inclusions were most abundant in astrocytes. No inclusions were detected in heart, liver, spleen or hilar lymph nodes. Based on their staining with luminescent conjugated oligothiophenes, we confirm that TMEM106B inclusions are amyloids. By in situ immunoelectron microscopy, TMEM106B assemblies were often found in structures resembling endosomes and lysosomes.

**Supplementary Information:**

The online version contains supplementary material available at 10.1186/s40478-024-01813-z.

## Introduction

Transmembrane protein 106B (*TMEM106B*) was first linked to neurodegenerative diseases, when sequence variations in its gene were identified as a risk factor for frontotemporal lobar degeneration with TDP-43 inclusions (FTLD-TDP), especially in individuals with granulin gene (*GRN*) mutations [42]. TMEM106B has also been implicated in neurodegenerative diseases other than ALS/FTLD-TDP [16, 31]. Moreover, genome-wide association studies have suggested a role for TMEM106B in age-associated phenotypes in the cerebral cortex, independently of disease, with increased neuronal density in cases with the protective haplotype [27, 34]. Outside the nervous system, TMEM106B has been identified as a receptor for SARS-CoV-2 cell entry in an angiotensin-converting enzyme 2 (ACE2)-independent manner [5, 37].

Electron cryo-microscopy (cryo-EM) has shown that amyloid filaments of TMEM106B can be found in human brains in an age-dependent manner [8, 14, 21, 23, 39]. They can be extracted from the brains of neurologically normal subjects and of individuals with neurodegenerative diseases, including those with filamentous inclusions made of amyloid-beta, tau, alpha-synuclein or TDP-43. TMEM106B inclusions do not co-localise with other protein aggregates [39, 49].

An increase in TMEM106B levels with age has been reported in carriers of the non-coding rs1990622 risk allele [19, 26]. The risk allele has also been associated with a higher number of TMEM106B aggregates [26, 28]. Conversely, the change from T185 to S185 (encoded by rs3173615) protects against FTLD-TDP [12, 30].

TMEM106B is a single-pass, type II transmembrane protein that is predominantly found in late endosomes and lysosomes; some protein may also localise to the plasma membrane [5, 16, 31]. TMEM106B is expressed ubiquitously, with highest levels in brain, heart, thyroid, testis and adrenal gland. In the brain, the expression of TMEM106B has been reported to be highest in neurons and oligodendrocytes and it has been implicated in myelination [51]. TMEM106B comprises a cytoplasmic portion (residues 1-96), followed by a transmembrane region (residues 97-117) and an intraluminal part (residues 118-274); the latter adopts a compact fibronectin type III domain with a seven-blade beta-sandwich fold [5]. The intraluminal portion carries glycosylation motifs at N145, N151, N164, N183 and N256 [25, 28].

TMEM106B is cleaved physiologically by an unknown protease that releases its C-terminal domain into the lysosomal lumen [6]. This generates a residual N-terminal fragment that is anchored to the lysosomal membrane. It is further cleaved by signal peptide peptidase-like 2A (SPPL2A) through intramembrane proteolysis, releasing an intracellular N-terminal domain into the cytosol and a small C-terminal domain into the lumen [32].

Release of the C-terminal domain of TMEM106B following cleavage at S120 is probably necessary for the formation of filaments, which extend from residues 120-254, suggesting that amino acids 255-274 are either unstructured or removed through a second cleavage. Here we show that such a cleavage appears likely, based on staining with an antibody specific for the C-terminus of TMEM106B (residues 263-274).

We also show that inclusions labelled by TMEM106B antibodies are fluorescent with amyloid dyes [4]; they were detected throughout central and peripheral nervous systems, where they were most abundant in glial cells. No inclusions were found in heart, liver, spleen or hilar lymph nodes of subjects with TMEM106B aggregates in the brain. By immunogold electron microscopy of cortical tissue sections TMEM106B filaments were located in vacuole-like structures and in structures resembling late endosomes and lysosomes.

## Materials and methods

### Cases

Table 1 and Supplementary Table 1 summarise characteristics of the cases investigated in this study. The cases used in the main figures are listed in Table 1. The 113 cases studied included 41 neurologically normal controls, 7 sporadic cases of  Alzheimer’s disease (AD), 2 familial AD cases (one with *PSEN1* and one with *APP* mutation), 15 cases of progressive supranuclear palsy (PSP), 6 cases of Pick’s disease (PiD), 2 cases of argyrophilic grain disease (AGD), 5  cases of frontotemporal dementia and parkinsonism linked to chromosome 17 caused by a *MAPT* mutation (FTDP-17T), 10 Parkinson’s disease (PD) cases (2 with *GBA* mutations, 8 sporadic), 2 cases of Parkinson’s disease dementia (PDD) (with *GBA* mutations), 2 cases of Lewy body dementia (LBD) (one with a *GBA* mutation), 2 cases of frontotemporal lobar degeneration with TDP-43 pathology (FTLD-TDP), 3 cases of multiple sclerosis, 2 cases of Sanfilippo A syndrome, 3  cases of neuroserpin, and one case each of juvenile-onset synucleinopathy (JOS), Alper’s disease, Down’s syndrome, Huntington’s disease, Friedreich’s ataxia, motor neuron disease (with *C9orf72* repeat expansion), vascular dementia, congophilic angiopathy, AD with atypical tauopathy, cerebellar degeneration, and dementia. In addition, we used brains from 7-month-old homozygous mice transgenic for human mutant P301S tau [1] and whole brains from 3- to 24-month-old control mice.

**Table 1:** Overview of cases

Case	Disease	Age (Years)	Gender	Mutation	Brain region; Peripheral organ	Cortical TMEM239 IHC score 0-4	References
1	Control	75	M	No	FL; Heart, Liver, Lymph nodes, Spleen	4	[39]
2	Control	25	M	No	FL; Heart, Liver, Lymph nodes, Spleen	0	[39]
3	PSP	78	M	No	FL, TL, OL, HPC, MB, BG, Pons, MD, CBL	4	
4	AD	90	M	No	FL, Spinal cord, DRG, Optic nerves	4	
5	Control	71	F	No	FL; Heart, Liver, Lymph nodes, Spleen	4	
6	Control	76	M	No	FL; Heart, Liver, Lymph nodes, Spleen	4	[39]
7	Sanfilippo A syndrome	11	M	*SGSH*	FL	0	[45]
8	JOS	15	F	*SNCA*	FL	0	[39]
9	Alper	25	M	No	FL, CBL	0	
10	Multiple sclerosis	33	F	No	FL	0	
11	Cerebellar degeneration	40	M	No	FL, BS, SC	0	
12	HD	44	M	*HTT*	FL, Thal	0	
13	Friedreich’s ataxia	49	M	No	FL, MD	0	
14	Down’s syndrome	49	M	Chromosome 21	FL, OL, MB	0	
15	FTDP-17 T	55	M	*MAPT (P301L)*	TL	4	[39]
16	PDD	57	M	*GBA*	FL	2	
17	AGD	74	M	No	HPC	4	
18	PiD	75	F	No	FL	4	
19	FTLD-TDP	75	M	No	FL	4	
20	PSP	78	F	No	FL, OL	4	
21	PD	82	F	No	FL, SN, HPC	4	
22	Control	98	M	No	PL, HPC	4	
23	Control	84	M	No	FL, BG	4	
24	Control	76	F	No	FL, BG	4	
25	Control	83	M	No	FL, BG	4	
26	Control	84	M	No	FL	4	[39]

*AD* Sporadic Alzheimer’s disease, *Alper* Alper’s disease, *AGD* Argryophilic grain disease, *Control* Neurologically normal individual, *JOS* Juvenile-onset synucleinopathy, *FTDP-17T* Familial frontotemporal dementia parkinsonism linked to chromosome 17 caused by *MAPT* mutations, *PD* Parkinson’s disease, *PiD* Pick’s disease, *PSP* Progressive supranuclear palsy. *BG* Basal ganglia, *CBL* Cerebellum, *DRG* Dorsal root ganglia, *FL* Frontal lobe, *HPC* Hippocampus, *MB* Midbrain, *MD* Medulla, *OL* Occipital lobe, *SN* Substantia nigra, *Thal* Thalamus, *TL* Temporal lobe. Semiquantitative TMEM239 IHC score: 0 (none), 1 (mild), 2 (moderate), 3 (abundant), 4 (severe)

### Antibodies

A rabbit polyclonal antibody (TMEM263) was raised to a synthetic peptide corresponding to residues 263-274 of human TMEM106B. To characterise its epitope, the TMEM106B C-terminal fragment (120-274) incorporated into pET3A was purchased from Genscript. The constructs lacking residues 239-250 (Δ239-250) or 263–274 (Δ263-274) were made using in vivo assembly [20]. Forward and reverse primers were obtained from Integrated DNA Technologies and designed to share 15-20 nucleotides of homologous region and 15-30 nucleotides for annealing to the template, flanking the region of deletion, with melting temperatures ranging from 58 to 65 °C. Before transformation, PCR products were treated with *DpnI*. Plasmids were transformed into *E. coli* BL21 cells, followed by expression of TMEM106B fragments and purification [39]. Antibody TMEM239 raised against residues 239-250 of human TMEM106B has been described [39]. A rabbit polyclonal antibody (TMEM193) was also raised to a synthetic peptide corresponding to residues 193-204 of human TMEM106B (Fig. S3). Antibody A303-439A, which was raised against amino acids 1-50 of human TMEM106B, was purchased from Bethyl Labs; antibody NBP1-91311, which was raised against residues 204-253 of murine TMEM106B, was purchased from Novus Biologicals; antibody AP22247b, which was raised against amino acids 218-252 of human TMEM106B, was purchased from Abcepta. Other antibodies were: NeuN antibody (Merck); GFAP antibody (Sigma Fine Chemicals); anti-APC antibody (CC1) (Merck); anti-Iba1/AIF antibody (Merck), and Cathepsin D (Santa Cruz Biotech).

### Immunohistochemistry

Single-labelling immunohistochemistry was carried out as described [39], with minor modifications. Specificity of the staining was established by antibody pre-adsorption (Supplementary Fig. 1). Following deparaffinization, formalin-fixed sections underwent heat-induced epitope retrieval (HIER) in Tris-EDTA buffer (10 mM Tris base, 1 mM EDTA, 0.05% Tween 20, pH 9) at 90 °C for 20 min. Other epitope retrieval methods tested included HIER in 10 mM citrate buffer (1.8 mM citric acid, 8.2 mM trisodium citrate, pH 6) at 90 °C for 20 min, and formic acid (87%) pre-treatment at room temperature (RT) for 10 min (Supplementary Fig. 1). This was followed by overnight incubation with primary antibody at 4 °C. Following washing in phosphate-buffered saline with 0.3% Triton X-100 (PBST), the sections were incubated with ImmPRESS-HRP polymer anti-rabbit detection antibody (Vector Laboratories) for 2 h at room temperature. Following washing with PBST, ImmPACT Vector SG substrate (peroxidase) was added to visualise the signal. Sections were counterstained with nuclear fast red (Vector Laboratories). For double-labelling immunohistochemistry, following completion of staining with a rabbit primary antibody, the sections were incubated overnight at 4 °C with the mouse primary antibody against the second antigen. Following washing in PBST, the sections were incubated with ImmPress-AP polymer anti-mouse detection antibody (Vector Laboratories) for 2 h at room temperature. Following washing, the second antigen was visualised with impact Vector Red substrate (alkaline phosphatase). Following dehydration, the sections were coverslipped with Entellan mounting medium (Merck). Images were acquired using a QImaging Retiga 2000 CCD camera mounted on an Olympus BX50 microscope.

The amount of TMEM106B aggregates was quantified by assessing TMEM239 IHC on cortical sections. A semiquantitave severity score was used in a five-graded scale as previously described [33]: absent (0), mild (1) (1–10 aggregates in a 10× field), moderate (2) (20–50 aggregates in a 10× field), abundant (3) (60–90 aggregates in a 10× field), and severe (4) (100 and more aggregates in a 10× field) TMEM239 positive staining. The person who performed the analysis was blinded to the disease groups and ages.

### Immunofluorescence and staining with amyloid dyes

Following incubation with primary antibodies overnight at 4 °C, the sections were washed in PBS and treated with TrueBlack lipofuscin autofluorescence quencher (Biotum) for 2 min at room temperature. Following washing in PBS, they were incubated with Alexa Fluor 568 and/or 647-conjugated secondary antibodies (Invitrogen, 1:250 in PBS) for 2 h at room temperature. Some sections were then labelled with either pentamer-formyl thiophene acetic acid (pFTAA, 3 µM), hepta-formyl thiophene acetic acid (hFTAA, 3 µM), HS-68 (3 µM) or Amytracker 540 (Ebba Biotech, 1 µM) in PBS or Thioflavin S (1% in distilled water) for 2 h at room temperature. Nuclei were counterstained with Hoechst dye (2.5 µg/ml in PBS, Sigma Fine Chemicals) for 15 min at room temperature. The sections were coverslipped with Fluoromount-G mounting medium (Southern Biotech). Images were captured on a Leica Stellaris 8 confocal microscope and processed using ImageJ.

### Immunogold *electron* microscopy of tissue sections

We used cerebral cortex from a 90-year-old male with AD (Table 1) and abundant TMEM106B inclusions. Tissue had been stored in formalin and one mm^3^ blocks were resected and fixed further in 2.5% glutaraldehyde with 2% paraformaldehyde overnight at 4 °C. Fixative was washed from the tissues with cacodylate buffer. Tissues were then treated with 1% osmium tetroxide and 1.5% potassium ferrocyanide in cacodylate for 1 h at room temperature. Samples were dehydrated in a graded ethanol series, incubated in acetone for 30 min and embedded in EPON resin for 48 h at 60 °C. Ultrathin Sections (70 nm) were cut using a diamond knife and placed on 200 mesh nickel grids (EM Sciences FCF200-Ni). The grids were floated on blocking solution (0.1% BSA in PBS) for 1 h at room temperature, followed by incubation with the TMEM239 primary antibody diluted 1:100 in blocking solution, overnight at 4 °C. Excess primary antibody was washed from the grids with wash buffer (0.01% BSA-C in PBS). The grids were floated on 10 nm immunogold goat-anti-rabbit-secondary antibody (EM Sciences 25109) diluted 1:40 in blocking solution for 2 h at room temperature. Excess secondary antibody was removed with wash buffer, followed by distilled water. The sections were contrasted for one min with UranyLess (EM Sciences), washed with water, blotted and dried in a vacuum dessicator for 1 h before imaging. All images were captured on a Tecnai 12 transmission electron microscope operated at 80 kV with a Hamamatsu Orca HR CCD camera.

### Tissue extraction and immunoblotting

Sarkosyl-insoluble material was extracted from brains and peripheral tissues, as described [39]. Briefly, tissues were homogenised in 20 volumes (v/w) extraction buffer consisting of 10 mM Tris–HCl, pH 7.5, 0.8 M NaCl, 10% sucrose and 1 mM EGTA. Homogenates were brought to 2% sarkosyl and incubated for 30 min at 37 °C. Following a 10 min centrifugation at 10,000 g, the supernatants were spun at 100,000 g for 20 min. The pellets were resuspended in 700 µl/g extraction buffer and centrifuged at 5000 g for 5 min. The supernatants were diluted threefold in 50 mM Tris-HCl, pH 7.5, containing 0.15 M NaCl, 10% sucrose and 0.2% sarkosyl, and spun at 166,000 g for 30 min. Sarkosyl-insoluble pellets were resuspended in 150 µl/g of 20 mM Tris–HCl, pH 7.4, containing 100 mM NaCl and sonicated twice for 10 min at 50% amplitude (QSonica). They were resolved on 12% Bis-Tris gels (Novex); TMEM239 antibody [39] was used at 1:2,000; A303-439A antibody at 1:500, and TMEM263 antibody at 1:1,000. To enhance the signal, the membranes were boiled in PBS for 10 min at 95 °C, as described [39].

## Results

### Cytoplasmic staining of TMEM106B by antibodies specific for its N- or C-termini

**Fig. 1:**
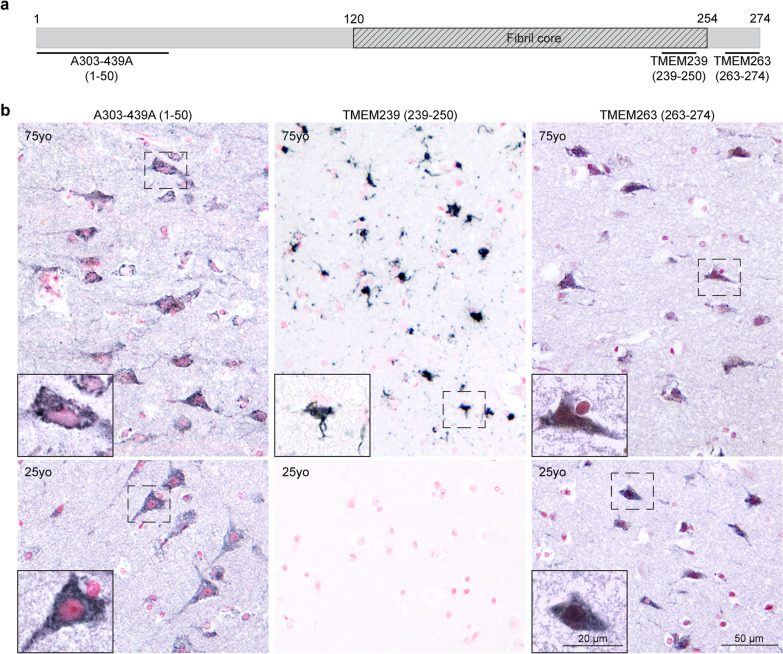
Immunohistochemical staining with anti-TMEM106B antibodies A303-439A, TMEM239 and TMEM263. **a**, Schematic of human TMEM106B, with the epitopes of anti-TMEM106B antibodies indicated. **b**, Staining of frontal cortex sections from neurologically normal individuals aged 75 (case 1) and 25 (case 2) years (yo = years-old). Nuclei are counterstained in red. Scale bars: 50 µm and 20 µm in the inset. Note similar cytoplasmic staining by A303-439A and TMEM263 in 25 yo and 75 yo individuals; TMEM239 stained inclusions in the 75 yo individual, with no staining in the 25 yo individual

In order to determine whether TMEM106B aggregates contained the full-length protein, we used antibody A303-439A [9, 22, 36, 49], which was raised against the N-terminal 50 amino acids of human TMEM106B, to stain tissue sections from the frontal cortex of a 25- and a 75-year-old neurologically normal individuals (Fig. 1, both cases were previously described [39]). Only diffuse cytoplasmic staining was observed. Similar results were obtained when we used antibody TMEM263, which was raised against a peptide corresponding to residues 263–274 of human TMEM106B which showed co-localization with endo-lysosomes (Fig. 1a, b; S6). No staining was observed following pre-adsorption of the antibody with TMEM106B (263–274) peptide (Fig. S1c). As previously reported [39], inclusions were identified using antibody TMEM239 (Fig. 1a, b; S6); they were also labelled by antibodies TMEM193, AP22247b and, as previously reported, antibody NB1-91311 [49] (Fig. S3). Staining of the frontal cortex from a 25-year-old individual lacking TMEM106B inclusions, similarly to the staining of the frontal cortex from the 75-year-old person with TMEM106B inclusions, showed diffuse cytoplasmic staining with A303-439A and TMEM263 (Fig. 1). A total of 28 brains from subjects aged 20–98 years old were stained with TMEM263, but no TMEM106B inclusions were detected by this antibody, and two representative cases are shown in Fig. 1. Antibodies A303-439A and TMEM263 also failed to recognise the 29 kDa band that was detected by antibody TMEM239 and is associated with the presence of TMEM106B aggregates (Fig. S2b).

### TMEM106B inclusions are present in central and peripheral nervous systems, but not in peripheral organs

**Fig. 2:**
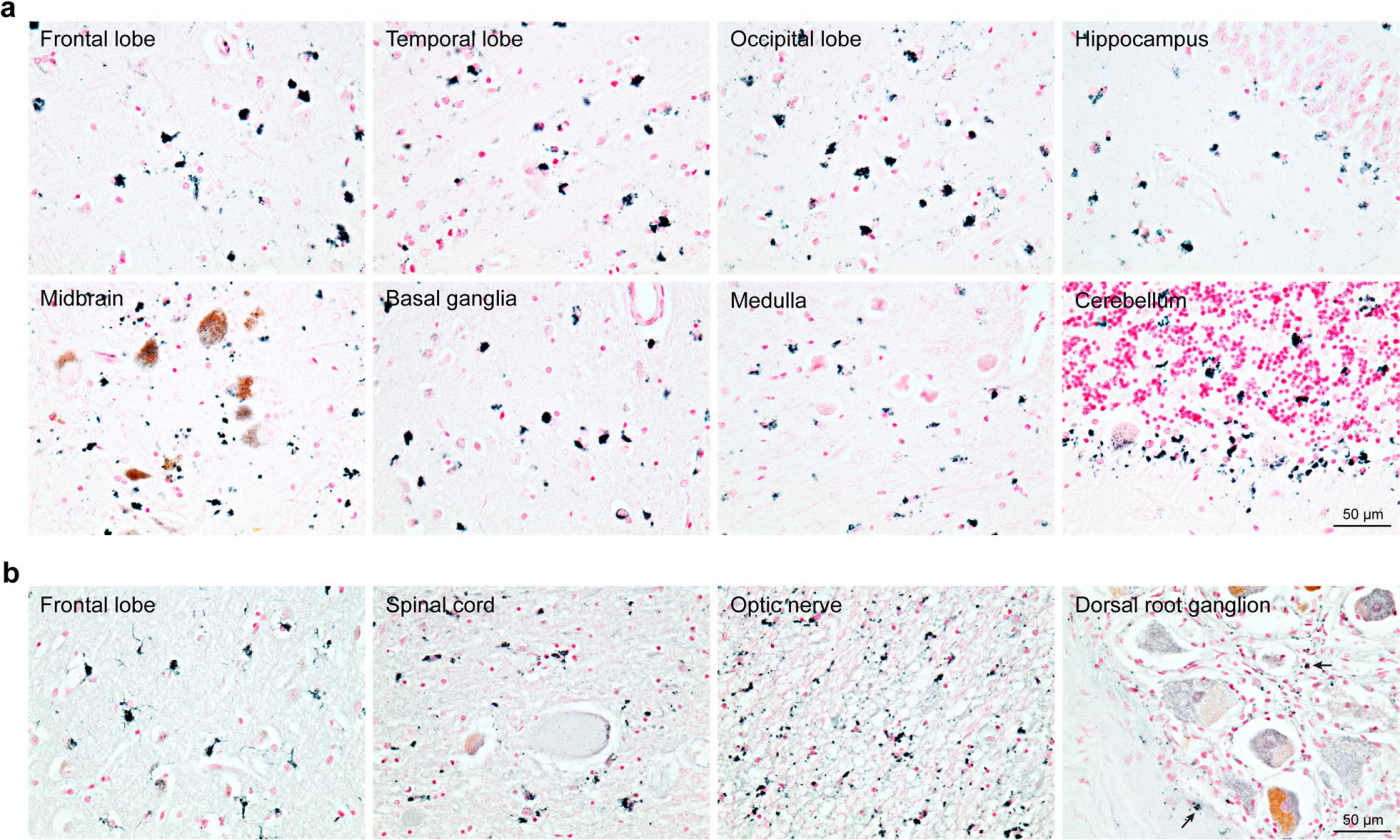
Presence of TMEM106B inclusions in central and peripheral nervous systems. **a**, Staining with antibody TMEM239 of different brain regions from an individual aged 78 years with progressive supranuclear palsy (case 3). Scale bar, 50 µm. **b**, Staining with antibody TMEM239 of brain, spinal cord and dorsal root ganglion sections from an individual aged 90 years with sporadic Alzheimer’s disease (case 4). Scale bar, 50 µm. Arrows in the dorsal root ganglion picture indicate TMEM106B aggregates

**Fig. 3:**
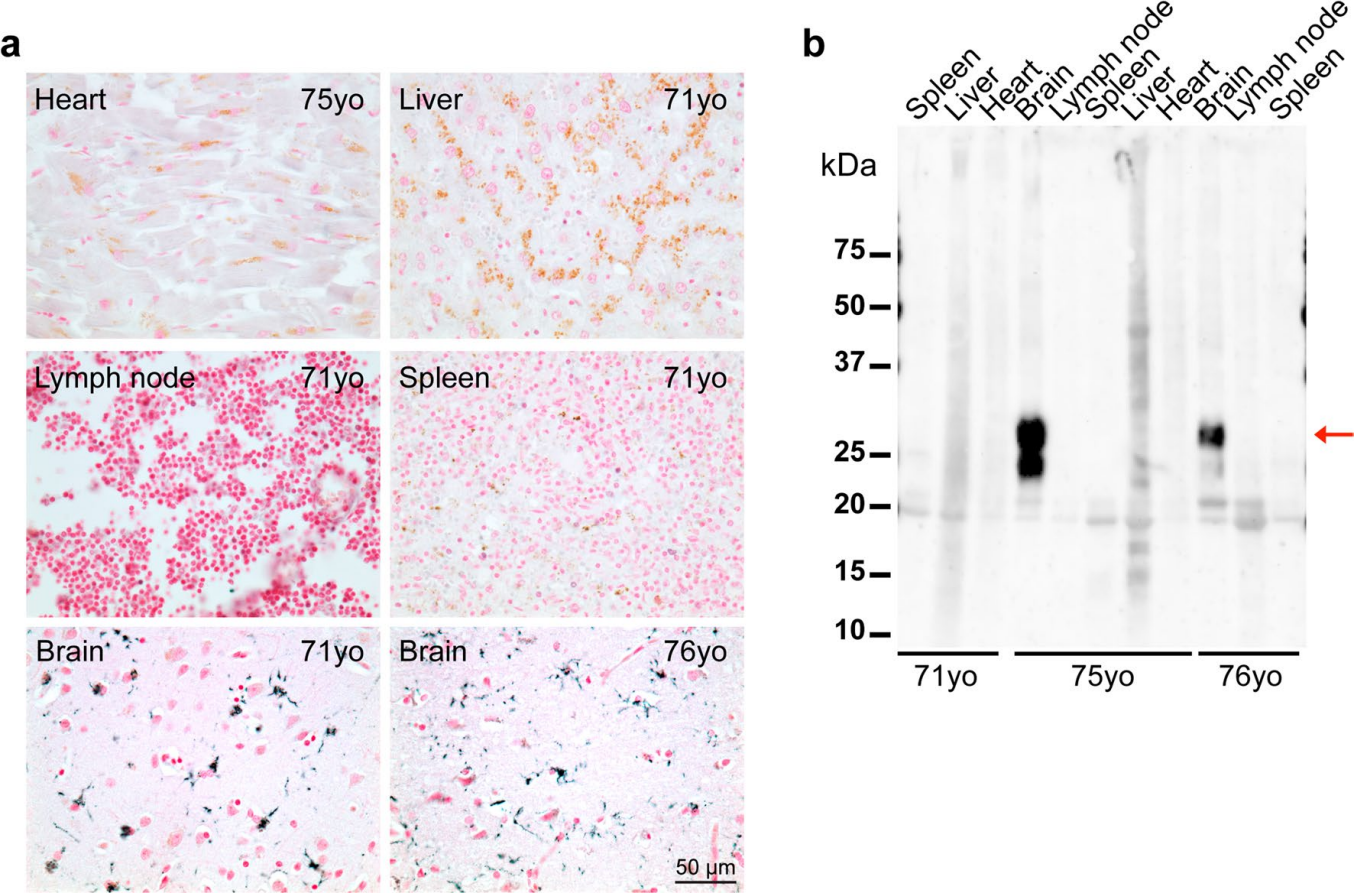
Absence of TMEM106B inclusions in peripheral organs. **a** Staining with TMEM239 of heart, liver, hilar lymph node, spleen and brain sections from neurologically normal individuals aged 71 (case 4), 75 (case 1) or 76 (case 6) years (yo = years-old). Nuclei are counterstained in red. Note the presence of TMEM106B inclusions in brain, but not in peripheral organs. Lipofuscin was observed as brown-coloured grainy material in some peripheral organs. Immunostaining of brain for the 75 yo subject is shown in Fig. 1. **b** Immunoblot analysis with TMEM239 of sarkosyl-insoluble extracts from heart, liver, hilar lymph node, spleen and brain from the same neurologically normal individuals aged 71, 75 or 76 years (yo = years-old) as in **a**. Note the presence of a 29-kDa band only in brain (red arrow). No brain tissue from the 71 yo individual was available for immunoblotting, but the presence of TMEM106B inclusions in brain is shown by immunostaining in **a**

Using antibody TMEM239, we used tissues from a 78-year-old individual with PSP and a 90-year-old individual with AD to investigate the presence of TMEM106B inclusions in central nervous system regions other than the frontal cortex (Fig. 2). As shown in Fig. 2, optic nerve, temporal cortex, occipital cortex, hippocampus, midbrain, basal ganglia, pons/medulla, cerebellum and spinal cord exhibited abundant TMEM106B inclusions. The same was also true of satellite cells in dorsal root ganglia, establishing the presence of TMEM106B inclusions in the peripheral nervous system (Fig. 2b). No inclusions were detected in heart, liver, spleen or hilar lymph nodes of neurologically normal individuals aged 71, 75 and 76 years with abundant TMEM106B inclusions in the brain (Fig. 3) or of a 49 year-old familial AD patient whose lung, gut, thyroid, and adrenal gland were also analysed (case 46, Supplementary Table 1). Young individuals were devoid of TMEM106B aggregates in all central and peripheral tissues examined (Supplementary Table 1).

### TMEM106B inclusions form in an age-dependent manner

**Fig. 4:**
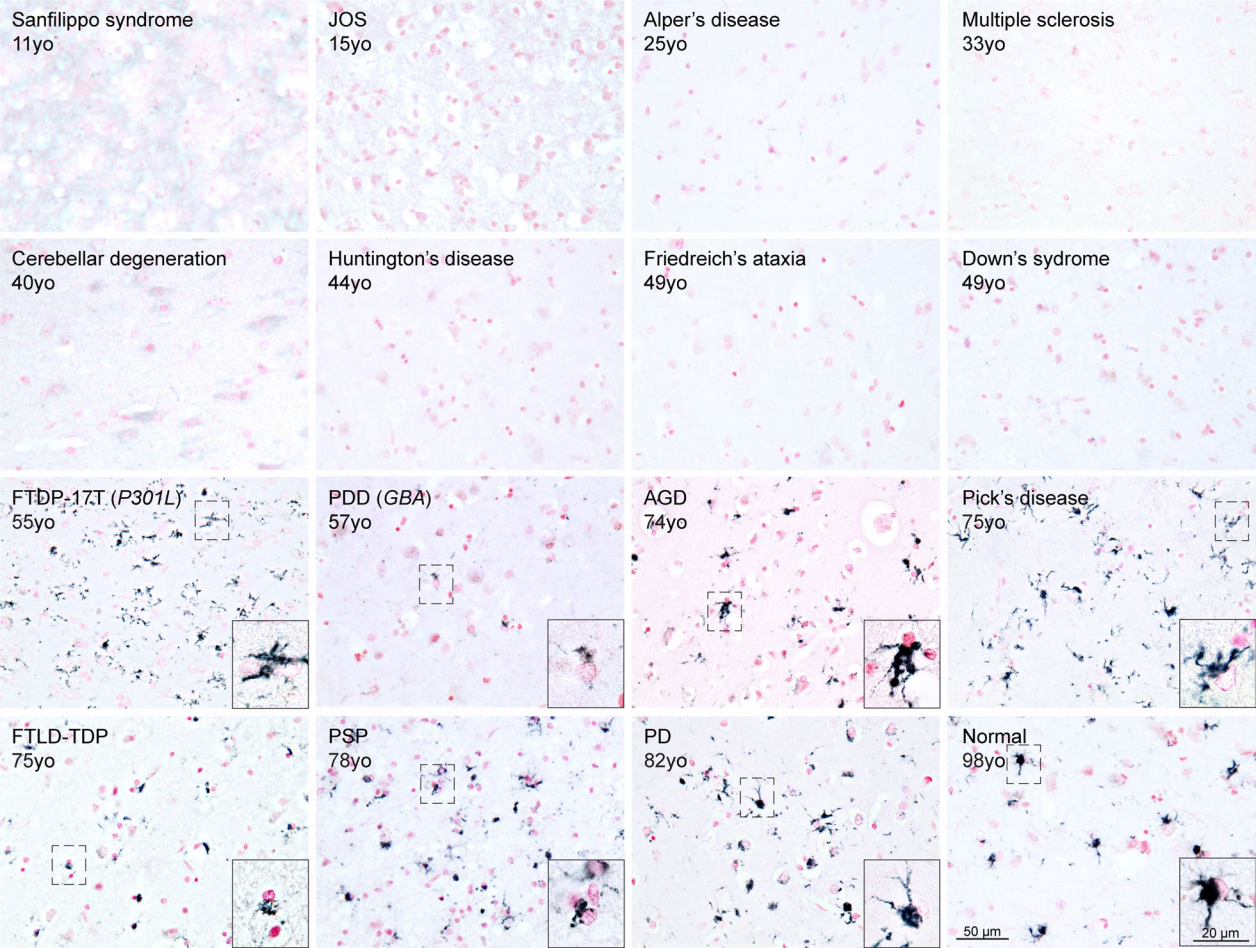
Age-dependent formation of TMEM106B inclusions, irrespective of the presence of disease. Staining with TMEM239 of frontal cortex sections from individuals with Sanfilippo syndrome (case 7), juvenile-onset synucleinopathy (JOS) (case 8), Alper’s disease (case 9), multiple sclerosis (case 10), cerebellar degeneration (case 11), Huntington’s disease (case 12), Friedreich’s ataxia (case 13), Down’s syndrome (case 14), frontotemporal dementia and parkinsonism linked to chromosome 17 with P301L mutation in *MAPT* (FTDP-17T) (case 15), Parkinson’s disease dementia (PDD) with a mutation in the glucocerebrosidase (*GBA*) gene (case 16), argyrophilic grain disease (AGD) (case 17), Pick’s disease (case 18), frontotemporal lobar degeneration with TDP-43 inclusions (FTLD-TDP) (case 19), progressive supranuclear palsy (PSP) (case 20), and Parkinson’s disease (PD) (case 21). Frontal cortex sections from a neurologically normal individual aged 98 years (case 22) were also used; yo = years-old. Scale bar, 50 µm and 20 µm (inset). TMEM106B inclusions were only detected in individuals older than 50 years

Previously, we [39] and others [8, 14, 22, 33, 34, 43, 49] have reported an association of TMEM106B aggregates with age. Here we extended this study by investigating the presence of TMEM106B inclusions in young individuals with a variety of diseases (Fig. 4; Table 1). No inclusions were found in the brains from individuals with Sanfilippo syndrome (aged 11y), juvenile-onset synucleinopathy (JOS) (aged 15y), Alper’s disease (aged 25y), multiple sclerosis (aged 33y), cerebellar degeneration (aged 40y), Huntington’s disease (aged 44y), Friedreich’s ataxia (aged 49y), Down’s syndrome (aged 49y) and familial Alzheimer’s disease (49y). TMEM106B inclusions were present in the brains from individuals with FTDP-17T (P301L mutation, aged 55y), PDD (aged 57y), AGD (aged 74y), PiD (aged 75y), FTLD-TDP (aged 75y), PSP (aged 78y), PD (aged 82y) and an aged control (98y). The cases shown in Fig. 4 are representative of all cases investigated, including those described in Supplementary Table 1. In Fig. S4 we show grading of the TMEM106B inclusions in conditions in which 5 or more cases were investigated.

So far, TMEM106B inclusions have been described in humans, we questioned whether they could also be present in aged mice. To this end we investigated their presence in the brains of 3- to 24-month-old wild-type and 7-month-old homozygous transgenic P301S tau mice with abundant tau pathology. No TMEM106B aggregates were detected (Fig. S5).

### TMEM106B inclusions are most abundant in astrocytes

**Fig. 5:**
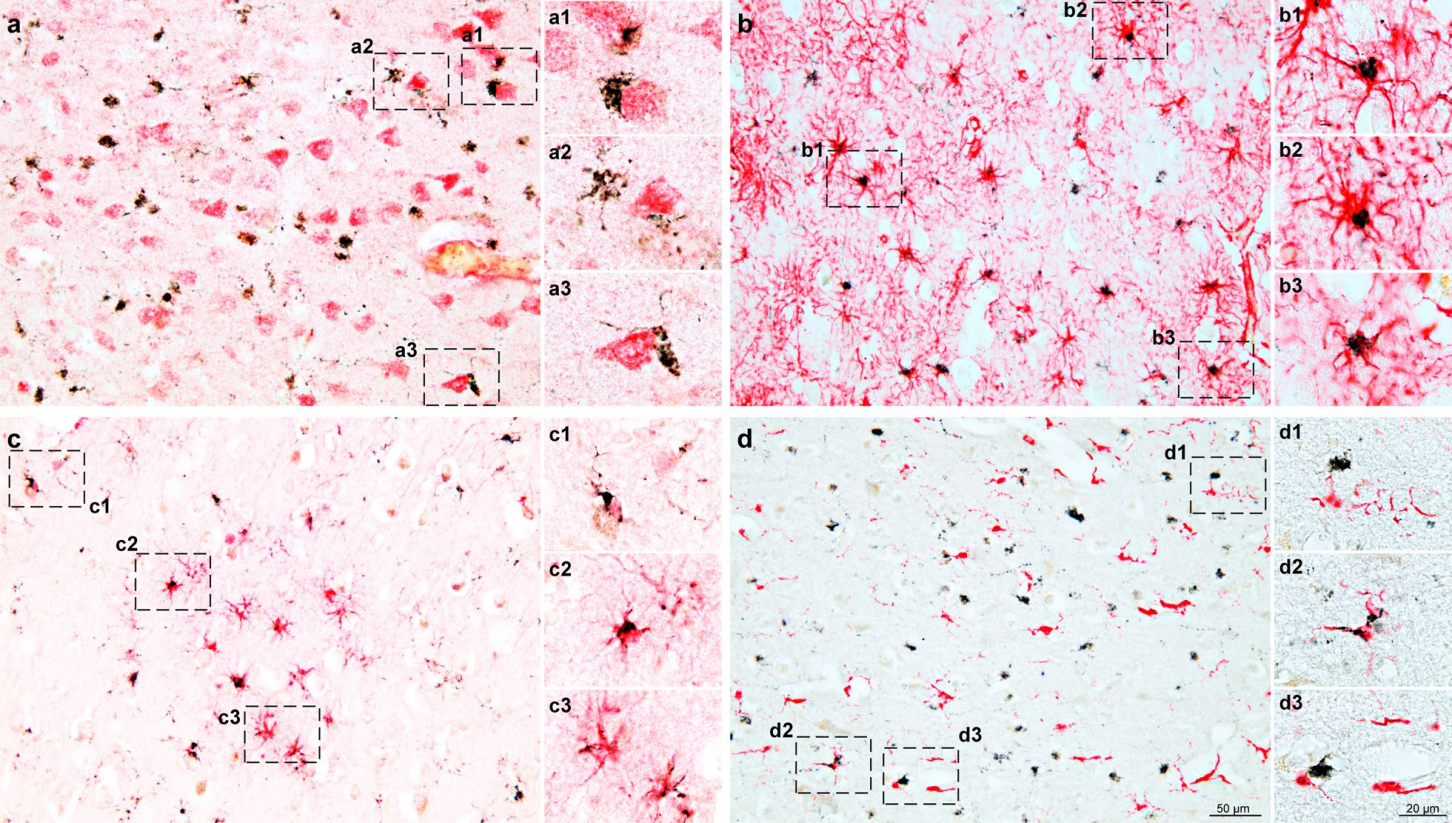
TMEM106B inclusions in brain cells. **a** Double-immunostaining of TMEM239 (black) and neuronal marker NeuN (red) of cortical sections from a neurologically normal individual aged 84 years (case 23); **a1**, **a2**, **a3**, close-up images of insets in **a**. TMEM106B inclusions were sometimes present in NeuN-positive nerve cells. **b** Double-immunostaining of TMEM239 (black) and astrocytic marker glial fibrillary acidic protein (GFAP) (red) of cortical sections from a neurologically normal individual aged 76 years (case 24); **b1**, **b2**, **b3**, close-up images of insets in **b**. Many TMEM106B inclusions were present in GFAP-positive astrocytes. **c** Double-immunostaining of TMEM239 (black) and oligodendrocytic marker APC/CC1 (red) of cortical sections from a neurologically normal individual aged 76 years (case 24); **c1**, **c2**, **c3**, close-up images of insets in **c**. TMEM106B inclusions were occasionally present in APC/CC1-positive oligodendrocytes. **d** Double-immunostaining of TMEM239 (black) and microglial marker Iba-1 (red) of cortical sections from a neurologically normal individual aged 84 years (case 23); **d1**, **d2**, **d3**, close-up images of insets in **d**. TMEM106B inclusions were occasionally present in Iba-1-positive microglia

We described earlier the presence of TMEM106B aggregates in glial cells [39].We now performed double-labelling immunohistochemistry of frontal cortex sections using antibody TMEM239 and antibodies against NeuN (neurons; Fig. 5a), GFAP (astrocytes; Fig. 5b), APC (oligodendrocytes; Fig. 5c) and Iba1 (microglia; Fig. 5d). As observed before [22, 28, 33, 49, 50] we found that TMEM106B inclusions were present predominantly in astrocytes (Fig. 5). Only some neurons, oligodendrocytes and microglia were double-labelled.

### TMEM106B inclusions contain amyloid filaments in situ

**Fig. 6:**
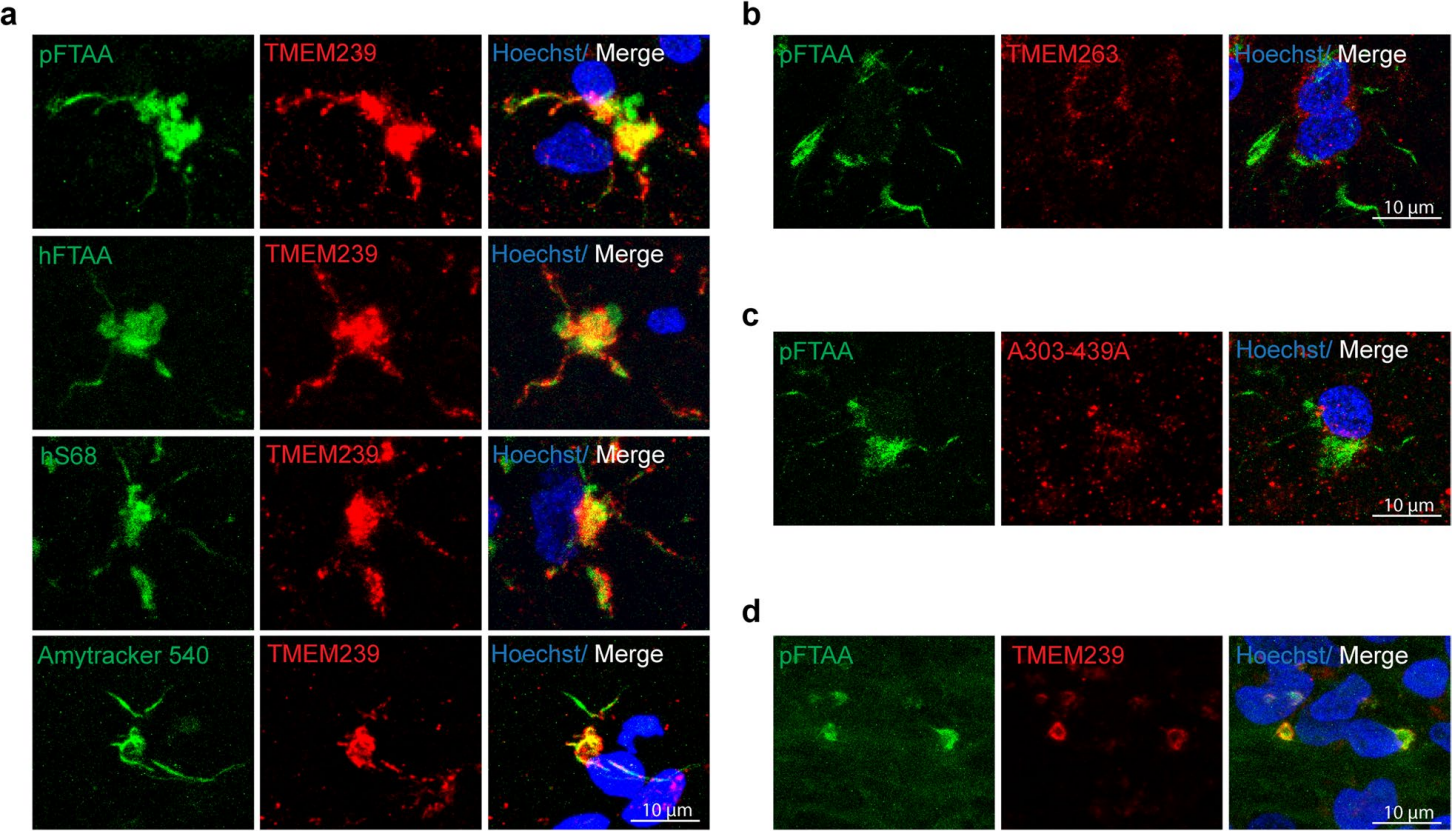
Double-labelling immunofluorescence of TMEM106B antibodies and luminescent conjugated oligothiophenes (LCOs), which are amyloid dyes. **a** Double-labelling immunofluorescence of sections from the frontal cortex of neurologically normal individuals aged 83 (case 25) and 84 (case 23) years with antibody TMEM239 (red) and LCOs pFTAA, hFTAA, HS68 and Amytracker 540 (green). Inclusions were labelled by both TMEM239 and LCOs (double labelling in yellow). **b**, **c** Double-labelling immunofluorescence of sections from the frontal cortex of a neurologically normal individual aged 84 years (case 26) using pFTAA and anti-TMEM106B antibodies TMEM263 (**b**) and A303-439A (**c**) (recognising TMEM106B C-terminus and N-terminus respectively). Inclusions were only labelled by pFTAA. **d** Double-labelling immunofluorescence of sections from the dorsal root ganglion of an individual aged 90 years with sporadic Alzheimer’s disease (case 4) with antibody TMEM239 (red) and pFTAA, (green). Inclusions were labelled by both TMEM239 and pFTAA (double labelling in yellow)

Amyloid dyes (Amytracker, hFTAA, pFTAA, HS68) labelled TMEM106B inclusions in central (Fig. 6a–c) and peripheral nervous systems (Fig. 6d). Antibodies A303-439A and TMEM263 did not colocalise with amyloid dyes (Fig. 6b,c). There were many lipofuscin-positive, TMEM106B-negative, cells in the brains from aged individuals. However, even when present in the same cells, TMEM106B inclusions and lipofuscin did not completely co-localise. Pre-adsorption of the antibody with TMEM106B (239-250) peptide gave no positive staining (Fig. S1b), supporting the specificity of staining.

### Immunogold electron microscopy of TMEM106B filaments in brain sections

**Fig. 7:**
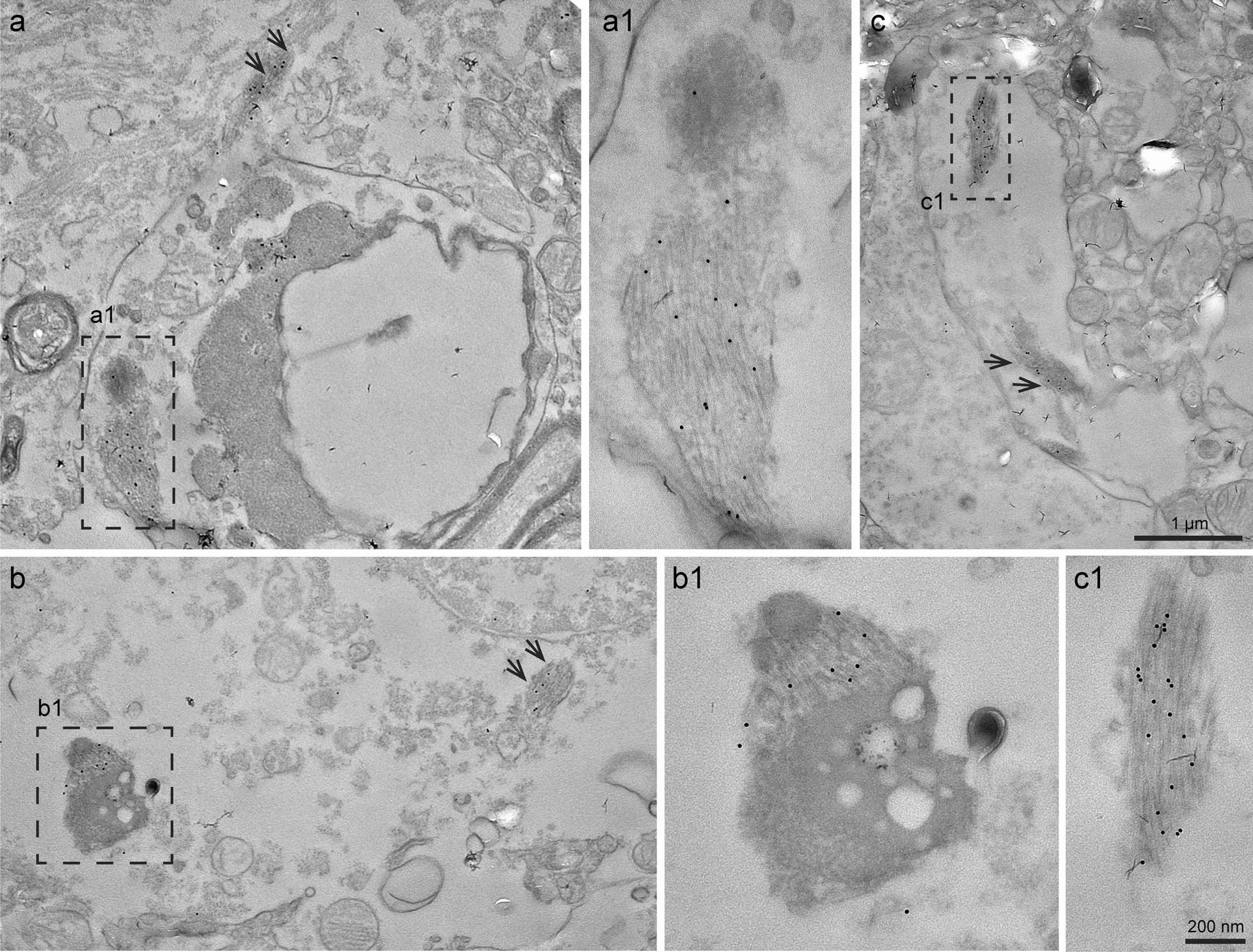
Immunogold electron microscopy on brain tissue sections. Immunogold EM on frontal cortex of an individual aged 90 years with Alzheimer’s disease (case 4) revealed the presence of bundles of filaments within the cell body and processes of cells, most likely astrocytes. The bundles of filaments decorated by gold particles were seen in association with dense osmiophilic structures with the morphological characteristics of secondary lysosomes. Bundles of filaments decorated by the gold particles, as well as the dense osmiophilic structures, may be present in vacuoles and were surrounded by a membrane. **a** Low power transversal view of a cell process containing immunogold labelled filaments enlarged in Box (**a1**). Adjacent to the transversal view of the cell process, another bundle of filaments decorated by gold particles is indicated by two arrows; **a1**, higher magnification of the smaller osmiophilic structure associated with the bundle of gold-decorated filament. **b** Low power view of a portion of a cell body. Part of the nucleus is seen in the upper right. Box (**b1**) shows an electron-dense osmiophilic structure with vacuoles that is associated with a bundle of filaments decorated by gold particles. Another bundle of filaments is indicated by two arrows; **b1** higher magnification of the electron-dense osmiophilic structure associated with filaments decorated by gold particles. **c** Low power view of a cell process containing two bundles of filaments, one in box (**c1**) and the other indicated by two arrows; c1, higher magnification of filaments seen in box (**c**). Scale bar in **c**, 1 µm (also for **a** and **b**) and in **c1**, 200 nm (also for **a1** and **b1**)

Immunogold electron microscopy revealed the presence of numerous bundles of filaments in the neuropil of the neocortex (Fig. 7). These filaments were seen in cell body (Fig. 7a,b) and processes (Fig. 7c) of cells that had the characteristics of astrocytes (nucleus without an evident nucleolus and sparse chromatin; little endoplasmic reticulum in the cytoplasm). Decorated filaments could be found within structures resembling secondary endo-lysosomes and in vacuoles (Fig. 7a). The immunogold labelling was specific in that gold particles were not observed in association with other cellular structures. In agreement, double immunofluorescence showed that some TMEM106B inclusions localised to lysosomes (Fig. S6a). No filament decoration was observed with antibody TMEM263.

## Discussion

Abundant filaments made of residues 120-254 of TMEM106B form in an age-dependent manner in human brains [33, 39, 43, 49], including those from individuals with various neurodegenerative conditions, such as AD, PD, DLB, FTDP-17T, FTLD-TDP, limbic-predominant age-related TDP-43 encephalopathy (LATE), pathological ageing and age-related tau astrogliopathy [3, 8, 14, 21, 23, 29, 39, 43, 49]. It remains to be seen if the formation of filaments of TMEM106B can influence the development and/or progression of disease. Lysosomal dysfunction is commonly observed during ageing and in neurodegenerative diseases [10] and it has been suggested that this may be a consequence of TMEM106B aggregation [24], although it is also possible that lysosomal dysfunction leads to TMEM106B aggregation. Interestingly, loss of TMEM106B has been shown to exacerbate TDP-43 and tau pathologies, as well as neurodegeneration in transgenic mice [13, 15, 17, 44, 52].

Many studies have focused on diseases with TDP-43 aggregates, for which the genetic association with TMEM106B was first shown [42]. Cases with the TMEM106B risk allele develop more TMEM106B inclusions [23, 28, 29, 33], although the influence on disease development remains uncertain. Unfortunately, the TMEM106B haplotype was not known in the cases investigated here, because they were obtained from brain banks as paraffin-embedded tissue sections with no DNA or fresh tissues available. However, we report that young people with either a lysosomal storage disorder, mitochondrial dysfunction or abundant protein aggregates, as in JOS, familial AD and Down syndrome, did not show TMEM106B aggregates. We cannot exclude that this could be the case of young individuals with *GRN* mutations for whom the TMEM106B haplotype is relevant for disease manifestation. This may become possible to investigate while the patient is still alive, given the recent finding that the PET ligand PBB3 recognises TMEM106B aggregates [50].

By immunohistochemistry, TMEM106B inclusions were most abundant in astrocytes, as reported previously [22, 28, 33, 49, 50], but they were also present in other cells, including neurons. In dorsal root ganglia, inclusions were most abundant in glial cells. This is interesting considering that previous studies reported higher TMEM106B protein expression in neurons and oligodendrocytes than astrocytes [16]. The origin of TMEM106B aggregates in astrocytes remains to be determined.

On immunoblots, inclusions ran as a 29-kDa band, as detected by antibodies specific for residues 239-250 of TMEM106B [28, 33, 39]. These antibodies also labelled TMEM106B inclusions by immunohistochemistry, as did antibody TMEM193, shown here, and previously described antibodies AP22247B and NBP1-91311 [49]. An antibody specific for residues 188-211 of TMEM106B has also been reported to label inclusions [22], as has an antibody against residues 191-206 [28].

TMEM106B is a transmembrane protein of 274 amino acids that is found in late endosomes/lysosomes [16]. Filament formation requires cleavage in the intraluminal domain between residues 119 and 120. Here we report that antibody TMEM263, which recognises the C-terminal part of recombinant TMEM106B on immunoblots (Fig. S2) and co-localises with lysosomes (Fig. S6), failed to recognise inclusions by immunohistochemistry, immunoblotting and immuno-EM, suggesting that residues 263-274 of TMEM106B are not part of the filaments.

TMEM263 gave diffuse cytoplasmic staining in brain cells, as did A303-439A, an antibody specific for the N-terminal region of TMEM106B. Similar diffuse staining for N-terminal and C-terminal antibodies has previously been reported [7, 9, 22, 36, 49]. Both antibodies gave similar staining, irrespective of age, as we showed in brain cells from 25- and 75-year-old individuals [39]. TMEM239 labelled inclusions in the brain from the 75-year-old individual [39], as well as in the brains from other aged subjects. However, TMEM106B aggregates were not found in aged mice.

TMEM106B filaments may lack a fuzzy coat, unlike filaments made of tau [18, 40], α-synuclein [38, 47], TDP-43 [2, 3] and amyloid-beta [46, 48]. This is also supported by mass spectrometry results that failed to detect C-terminal peptides, including residues 255-262, in brain extracts [3, 14]. Lack of a fuzzy coat could influence the spreading and toxicity of assembled TMEM106B [41]. Whether TMEM106B filaments can exhibit prion-like properties is not known. Not only may this require two cleavages, but the reported stability of TMEM106B filaments [23, 35] may limit their spreading since the prion-like properties are inversely related to the stability of assembled proteins [11].

The formation of TMEM106B inclusions in the brain is age-dependent and disease-independent [39, 43]. As shown here, sensory ganglia and spinal cord also contained TMEM106B aggregates, while no inclusions were detected in heart, liver, spleen or hilar lymph nodes of subjects with brain aggregates. Moreover, TMEM106B inclusions were not present in the central nervous system of young individuals, even in the presence of disease.

By immunoelectron microscopy of cortical sections from a case of AD with abundant TMEM106B inclusions, we found filaments decorated by antibody TMEM239 that were closely associated with secondary lysosomes and vacuoles. This suggests that TMEM106B may assemble first in the organelles that normally express the native protein or that assembly can occur after escaping the damaged lysosomes.

In conclusion, we show that TMEM106B filaments are stained by amyloid dyes and confirm that they are predominantly present in non-neuronal cells. TMEM106B aggregates were not present in several peripheral human organs; similarly, they were also absent from wild-type and transgenic mouse brains. By immunogold-EM aggregates were found to be associated with lysosomes and vacuolar structures. By light microscopy, the inclusions were labelled by an antibody against the core region of TMEM106B filaments, but not by an antibody against the C-terminus of TMEM106B. It remains to be determined what roles these inclusions may have in the brains of aged human individuals.

### Supplementary Information

Supplementary Figure 1. Immunohistochemical staining optimisation and specificity. (a), Several antigen retrieval methods were tested and provided different immunostaining results with TMEM239 antibody using medulla sections of a Parkinson’s disease case aged 73 years (case 39). No staining was observed after heat-induced epitope retrieval (HIER) with citrate buffer or without pretreatment, whereas formic acid treatment or HIER with Tris-EDTA buffer resulted in strong staining. Nuclei were counterstained in red. (b, c), Immunostaining with antibodies TMEM239 (b), and TMEM263 (c) of adjacent cortical sections from (b) a 78-year-old (case 3) and (c) a 73-year-old (case 70) individual with progressive supranuclear palsy. Following pre-adsorption of the antibodies with their respective immunogens, all the stainings were abolished.

Supplementary Figure 2. Characterisation of antibody TMEM263 by immunoblotting. (a), TMEM106B (120-274, WT) and TMEM106B (120-262, Δ263-274) were expressed in E. coli. The pellets from 1 ml bacterial cultures were used for immunoblotting with antibodies TMEM239 and TMEM263. Red arrows point to TMEM106B bands, whereas red asterisks indicate non-specific binding. Note that TMEM239 labelled both TMEM106B (120-274, WT) and TMEM106B (120-262, Δ263-274), whereas TMEM263 only labelled TMEM106B (120-274). (b), Immunoblots of sarkosyl soluble (Sark. sol) and sarkosyl-insoluble (Sark. insol) fractions from the frontal cortex of 58 yo neurologically normal individual (case 99) stained with antibodies A303-439A, TMEM239, and TMEM263. A 29kDa band is observed with the TMEM239 antibody in the sarkosyl-insoluble fraction, this band was not recognised by TMEM263, and A303-439A antibodies supporting the absence of the N-terminus and C-terminus of TMEM106B in the aggregates. The antibodies recognised similar bands in the sarkosyl-soluble fraction.

Supplementary Figure 3. Staining of TMEM106B inclusions with antibodies TMEM193, AP22247b and NBP1-91311. (a), Schematic of human TMEM106B, with the epitopes of anti-TMEM106B antibodies indicated. (b), Staining of frontal cortex sections from neurologically normal controls, aged 25 (case 2), 75 (case 1) and 76 (case 6) (yo=years-old). Nuclei are counterstained in red. Scale bar, 50 µm and 20 µm (insets). Note the presence of TMEM106B inclusions in the brains of individuals aged 75 and 76 and the absence of staining in the brain from the 25-year-old individual.

Supplementary Figure 4. Presence of TMEM106B inclusions in relation to age in brain of healthy and diseased subjects. TMEM106B inclusion burden determined by TMEM239 IHC, and scored semiquantitavely by a researcher blinded to disease status and age. (a), TMEM239 IHC scores versus age in neurologically normal individuals and (b), in diseased individuals. Only disease cohorts which had at least 5 individuals analysed are indicated (b). In neurologically normal individuals as well as subjects affected by disease the amount of TMEM106B inclusions correlated with age. Each circle represents an individual in the cohort, and colour of the circles represents the TMEM239 IHC score. Semi-quantitative IHC score: 0 (white), 1 (green), 2 (blue), 3 (yellow), 4 (red).

Supplementary Figure 5. No TMEM106B inclusions were detected in the brains of young and aged mice. Staining with TMEM239 of representative cerebral cortex sections (treated with formic acid) from C57BL6/J mice aged 3 months and 18 months. Nuclei are counterstained in red. Scale bar, 100 µm.

Supplementary Figure 6. Double-labelling immunofluorescence of TMEM106B antibodies and a lysosomal marker. (a), Double-labelling immunofluorescence of sections from the frontal cortex of neurologically normal individuals aged 72 (case 107) with antibody TMEM239 (green) and lysosomal marker cathepsin D (red). Inclusions were labelled by TMEM239. Some TMEM106B inclusions co-localised with cathepsin D staining (double labelling in yellow) indicating their lysosomal localization. (b), Double-labelling immunofluorescence of sections from the frontal cortex of a neurologically normal individual aged 76 (case 108) with antibody TMEM263 (green) and lysosomal marker cathepsin D (red). TMEM263 staining co-localised with lysosomes (double labelling in yellow).

Supplementary Table 1.

## References

[1] Allen B, Ingram E, Takao M, Smith MJ, Jakes R, Virdee K et al (2002) Abundant tau filaments and nonapoptotic neurodegeneration in transgenic mice expressing human P301S tau protein. J Neurosci 22:9340–9351 12417659 10.1523/JNEUROSCI.22-21-09340.2002 PMC6758022

[2] Arseni D, Hasegawa M, Murzin AG, Kametani F, Arai M, Yoshida M et al (2022) Structure of pathological TDP-43 filaments from ALS with FTLD. Nature 601:139–143 34880495 10.1038/s41586-021-04199-3 PMC7612255

[3] Arseni D, Chen R, Murzin AG, Peak-Chew SY, Garringer HJ, Newell KL et al (2023) TDP-43 forms amyloid filaments with a distinct fold in type A FTLD-TDP. Nature 620:898–903 37532939 10.1038/s41586-023-06405-w PMC10447236

[4] Åslund A, Sigurdson CJ, Klingstedt T, Grathwohl S, Bolmont T, Dickstein DL et al (2009) Novel pentameric thiophene derivatives for in vitro and in vivo imaging of a plethora of protein aggregates in cerebral amyloidoses. ACS Chem Biol 4:673–683 19624097 10.1021/cb900112v PMC2886514

[5] Baggen J, Jacquemyn M, Persoons L, Vanstreels E, Pye VE, Wrobel AG et al (2023) TMEM106B is a receptor mediating ACE2-independent SARS-CoV-2 cell entry. Cell 186:3427–3442 37421949 10.1016/j.cell.2023.06.005 PMC10409496

[6] Brady OA, Zheng Y, Murphy K, Huang M, Hu F (2013) The frontotemporal lobar degeneration risk factor, TMEM106B, regulates lysosomal morphology and function. Hum Mol Genet 22:685–695 23136129 10.1093/hmg/dds475 PMC3554197

[7] Busch JI, Martinez-Lage M, Ashbridge E, Grossman M, Van Deerlin VM, Hu F et al (2013) Expression of TMEM106B, the frontotemporal lobar degeneration-associated protein, in normal and diseased human brain. Acta Neuropathol Commun 1:36 24252750 10.1186/2051-5960-1-36 PMC3893524

[8] Chang A, Xiang X, Wang J, Lee C, Arakhamia T, Simjanoska M et al (2022) Homotypic fibrillization of TMEM106B across diverse neurodegenerative diseases. Cell 185:1346–1355 35247328 10.1016/j.cell.2022.02.026 PMC9018563

[9] Chen-Plotkin AS, Unger TL, Gallagher MD, Bill E, Kwong LK, Volpicelli-Daley L et al (2012) *TMEM106B*, the risk gene for frontotemporal dementia, is regulated by the miRNA-132/212 cluster and affects progranulin pathways. J Neuroscience 32:11213–11227 22895706 10.1523/JNEUROSCI.0521-12.2012 PMC3446826

[10] Colacurcio DJ, Nixon RA (2016) Disorders of lysosomal acidification. The emerging role of v-ATPase in aging and neurodegenerative disease. Ageing Res Rev 32:75–88 27197071 10.1016/j.arr.2016.05.004 PMC5112157

[11] Colby DW, Giles K, Legname G, Wille H, Baskakov IV, DeArmond SJ et al (2009) Design and construction of diverse mammalian prion strains. Proc Natl Acad Sci USA 106:20417–20422 19915150 10.1073/pnas.0910350106 PMC2787151

[12] Cruchaga C, Graff C, Chiang HH, Wang J, Hinrichs AL, Spiegel N et al (2011) Association of TMEM106B gene polymorphism with age at onset in granulin mutation carriers and plasma granulin protein levels. Arch Neurol 68:581–586 21220649 10.1001/archneurol.2010.350 PMC3090529

[13] Edwards GA, Wood CA, He Y, Nguyen Q, Kim PJ, Gomez-Gutierez R et al (2024) TMEM106B coding variant is protective and deletion detrimental in a mouse model of tauopathy. Acta Neuropathol 147:61 38526616 10.1007/s00401-024-02701-5 PMC12313335

[14] Fan Y, Zhao Q, Xia W, Tao Y, Yu W, Chen M et al (2022) Generic amyloid fibrillation of TMEM106B in patient with Parkinson’s disease dementia and normal elders. Cell Res 32:585–588 35477998 10.1038/s41422-022-00665-3 PMC9160068

[15] Feng T, Mai S, Roscoe JM, Sheng RR, Ullah M, Zhang J et al (2020) Loss of TMEM106B and PGRN leads to severe lysosomal abnormalities and neurodegeneration in mice. EMBO Rep 21:e50219 32852886 10.15252/embr.202050219 PMC7534636

[16] Feng T, Lacrampe A, Hu F (2021) Physiological and pathological functions of TMEM106B: a gene associated with brain aging and multiple brain disorders. Acta Neuropathol 141:327–339 33386471 10.1007/s00401-020-02246-3 PMC8049516

[17] Feng T, Du H, Yang C, Wang Y, Hu F (2024) Loss of TMEM106B exacerbates Tau pathology and neurodegeneration in PS19 mice. Acta Neuropathol 147:62 38526799 10.1007/s00401-024-02702-4 PMC11924916

[18] Fitzpatrick AWP, Falcon B, He S, Murzin AG, Murshudov G, Garringer HJ et al (2017) Cryo-EM structures of tau filaments from Alzheimer’s disease. Nature 547:185–190 28678775 10.1038/nature23002 PMC5552202

[19] Gallagher MD, Posavi M, Huang P, Unger TL, Berlyand Y, Gruenewald AL et al (2017) A dementia-associated risk variant near TMEM106B alters chromatin architecture and gene expression. Am J Hum Genet 101:643–653 29056226 10.1016/j.ajhg.2017.09.004 PMC5673619

[20] Garcia-Nafría J, Watson JF, Greger IH (2016) IVA cloning: a single-tube universal cloning system exploiting in vivo assembly. Sci Rep 6:27459 27264908 10.1038/srep27459 PMC4893743

[21] Hoq MR, Bharath SR, Hallinan GI, Fernandez A, Vago FS, Ozcan KA et al (2023) Cross-β helical filaments of tau and TMEM106B in gray and white matter of multiple system tauopathy with presenile dementia. Acta Neuropathol 145:707–710 36952000 10.1007/s00401-023-02563-3 PMC10119215

[22] Ishikawa R, Yamazaki Y, Nakamori M, Takahashi T, Maruyama H (2023) Antibody-recognizing residues 188–211 of TMEM106B exhibit immunohistochemical reactivity with the TMEM106B C-terminal fragment. Front Neurosci 17:1250547 37937069 10.3389/fnins.2023.1250547 PMC10626444

[23] Jiang YX, Cao Q, Sawaya MR, Abskharon R, Ge P, DeTure M et al (2022) Amyloid fibrils in FTLD-TDP are composed of TMEM106B and not TDP-43. Nature 605:304–309 35344984 10.1038/s41586-022-04670-9 PMC9844993

[24] Jiao HS, Yuan P, Yu JT (2023) TMEM106B aggregation in neurodegenerative diseases: linking genetics to function. Mol Neurodegen 18:54 10.1186/s13024-023-00644-1 PMC10413548 37563705

[25] Lang CM, Fellerer K, Schwenk BM, Kuhn PH, Kremmer E, Edbauer D et al (2012) Membrane orientation and subcellular localization of transmembrane protein 106B (TMEM106B), a major risk factor for frontotemporal lobar degeneration. J Biol Chem 287:19355–19365 22511793 10.1074/jbc.M112.365098 PMC3365973

[26] Lee JY, Harney DJ, Teo JD, Kwok JB, Sutherland GT, Larance M et al (2023) The major *TMEM106B* dementia risk allele affects TMEM106B protein levels, fibril formation, and myelin lipid homeostasis in the ageing human hippocampus. Mol Neurodeg 18:63 10.1186/s13024-023-00650-3 PMC10510131 37726834

[27] Li Z, Farias FHG, Dube U, Del-Aguila JL, Mihindukulasuriya KA, Fernandez MV et al (2020) The TMEM106B FTLD-protective variant, rs1990621, is also associated with increased neuronal proportion. Acta Neuropathol 139:45–61 31456032 10.1007/s00401-019-02066-0 PMC6942643

[28] Marks JD, Ayuso VE, Carlomagno Y, Yue M, Todd TW, Hao Y et al (2024) TMEM106B core deposition associates with TDP-43 pathology and is increased in risk SNP carriers for frontotemporal dementia. Sci Transl Med 16:eadf9735 38232138 10.1126/scitranslmed.adf9735 PMC10841341

[29] Neumann M, Perneel J, Cheung S, van den Broeck M, Nygaard H, Hsiung GYR et al (2023) Limbic-predominant age-related TDP-43 proteinopathy (LATE-NC) is associated with abundant TMEM106B pathology. Acta Neuropathol 146:163–166 37171635 10.1007/s00401-023-02580-2

[30] Nicholson AM, Finch NA, Wojtas A, Baker MC, Perkerson RB, Castanedes-Casey M et al (2013) TMEM106B p. T185 regulates TMEM106B protein levels: Implications for frontotemporal dementia. J Neurochem 126:781–791 23742080 10.1111/jnc.12329 PMC3766501

[31] Nicholson AM, Rademakers R (2016) What we know about TMEM106B in neurodegeneration. Acta Neuropathol 132:639–651 27543298 10.1007/s00401-016-1610-9 PMC5074873

[32] Perneel J, Rademakers R (2022) Identification of TMEM106B amyloid fibrils provides an updated view of TMEM106B biology in health and disease. Acta Neuropathol 144:807–819 36056242 10.1007/s00401-022-02486-5 PMC9547799

[33] Perneel J, Neumann M, Heeman B, Cheung S, Van den Broeck M, Wynants S et al (2023) Accumulation of TMEM106B C-terminal fragments in neurodegenerative disease and aging. Acta Neuropathol 145:285–302 36527486 10.1007/s00401-022-02531-3

[34] Rhinn H, Abeliovich A (2017) Differential aging analysis in human cerebral cortex identifies variants in TMEM106B and GRN that regulate aging phenotypes. Cell Syst 4:404–415 28330615 10.1016/j.cels.2017.02.009

[35] Sawaya MR, Hughes MP, Rodriguez JA, Riek R, Eisenberg DS (2021) The expanding amyloid family: structure, stability, function, and pathogenesis. Cell 184:4857–4873 34534463 10.1016/j.cell.2021.08.013 PMC8772536

[36] Satoh J, Kino Y, Kawana N, Yamamoto Y, Ishida T, Saito Y et al (2014) TMEM106B expression is reduced in Alzheimer’s disease brains. Alz Res Therapy 6:17 10.1186/alzrt247 PMC4055042 24684749

[37] Schneider WM, Luna JM, Hoffmann HH, Sánchez-Rivera FJ, Leal AA, Ashbrook AW et al (2021) Genome-scale identification of SARS-Cov-2 and pan-coronavirus host factor networks. Cell 184:120–132 33382968 10.1016/j.cell.2020.12.006 PMC7796900

[38] Schweighauser M, Shi Y, Tarutani A, Kametani F, Murzin AG, Ghetti B et al (2020) Structures of α-synuclein filaments from multiple system atrophy. Nature 585:464–469 32461689 10.1038/s41586-020-2317-6 PMC7116528

[39] Schweighauser M, Arseni D, Bacioglu M, Huang M, Lövestam S, Shi Y et al (2022) Age-dependent formation of TMEM106B amyloid filaments in human brains. Nature 605:310–314 35344985 10.1038/s41586-022-04650-z PMC9095482

[40] Shi Y, Zhang W, Yang Y, Murzin AG, Falcon B, Kotecha A et al (2021) Structure-based classification of tauopathies. Nature 598:359–363 34588692 10.1038/s41586-021-03911-7 PMC7611841

[41] Ulamec SM, Brockwell DJ, Radford SE (2020) Looking beyond the core: the role of flanking regions in the aggregation of amyloidogenic peptides and proteins. Front Neurosci 14:611285 33335475 10.3389/fnins.2020.611285 PMC7736610

[42] Van Deerlin VM, Sleiman PM, Martinez-Lage M, Chen-Plotkin A, Wang LS, Graff-Radford NR et al (2010) Common variants at 7p21 are associated with frontotemporal lobar degeneration with TDP-43 inclusions. Nature Genet 42:234–239 20154673 10.1038/ng.536 PMC2828525

[43] Vicente CT, Perneel J, Wynants S, Heeman B, van den Broeck M, Baker M et al (2023) C-terminal TMEM106B fragments in human brain correlate with disease-associated *TMEM106B* haplotypes. Brain 146:4055–4064 37100087 10.1093/brain/awad133 PMC10545506

[44] Werner G, Damme M, Schludi M, Gnörich J, Wind K, Fellerer K et al (2020) Loss of TMEM106B potentiates lysosomal and FTLD-like pathology in progranulin-deficient mice. EMBO Rep 21:e50241 32929860 10.15252/embr.202050241 PMC7534633

[45] Winder-Rhodes SE, Garcia-Reitböck P, Ban M, Evans JR, Jacques TS, Kemppinen A et al (2012) Genetic and pathological links between Parkinson’s disease and the lysosomal disorder Sanfilippo syndrome. Mov Disord 27:312–315 22102531 10.1002/mds.24029

[46] Yang Y, Arseni D, Zhang W, Huang M, Lövestam S, Schweighauser M et al (2022) Cryo-EM structures of amyloid-β 42 filaments from human brains. Science 375:167–172 35025654 10.1126/science.abm7285 PMC7612234

[47] Yang Y, Shi Y, Schweighauser M, Zhang X, Kotecha A, Murzin AG et al (2022) Structures of α-synuclein filaments from human brains with Lewy pathology. Nature 610:791–795 36108674 10.1038/s41586-022-05319-3 PMC7613749

[48] Yang Y, Zhang W, Murzin AG, Schweighauser M, Huang M, Lövestam S et al (2023) Cryo-EM structures of amyloid-β filaments with the Arctic mutation (E22G) from human and mouse brains. Acta Neuropathol 145:325–333 36611124 10.1007/s00401-022-02533-1 PMC9925504

[49] Zhao W, Fan Y, Zhao Q, Fan Z, Zhao J, Yu W et al (2024) Tracing TMEM106B fibril deposition in aging and Parkinson’s disease with dementia brains. Life Med 3:lnae011 10.1093/lifemedi/lnae011 PMC11749594 39872397

[50] Zhao Q, Fan Y, Zhao W, Ni Y, Tao Y, Bian J et al (2024) A Tau PET tracer PBB3 binds to TMEM106B amyloid fibril in brain. Cell Discov 10:50 38744856 10.1038/s41421-024-00674-z PMC11094151

[51] Zhou X, Nicholson AM, Ren Y, Brooks M, Jiang P, Zuberi A et al (2020) Loss of TMEM106B leads to myelination deficits: implications for frontotemporal dementia treatment strategies. Brain 143:1905–1919 32504082 10.1093/brain/awaa141 PMC7296855

[52] Zhou X, Brooks M, Jiang P, Koga S, Zuberi AR, Baker MC et al (2020) Loss of Tmem106b exacerbates FTLD pathologies and causes motor deficits in progranulin-deficient mice. EMBO Rep 21:e50197 32761777 10.15252/embr.202050197 PMC7534638

