# Correction: Cleaved TMEM106B forms amyloid aggregates in central and peripheral nervous systems

**DOI:** 10.1186/s40478-024-01842-8

**Published:** 2024-08-14

**Authors:** Mehtap Bacioglu, Manuel Schweighauser, Derrick Gray, Sofia Lövestam, Taxiarchis Katsinelos, Annelies Quaegebeur, John van Swieten, Zane Jaunmuktane, Stephen W. Davies, Sjors H. W. Scheres, Michel Goedert, Bernardino Ghetti, Maria Grazia Spillantini

**Affiliations:** 1 Department of Clinical Neurosciences, University of Cambridge, Cambridge, UK; 2 Medical Research Council Laboratory of Molecular Biology, Cambridge, UK; 3 IUSM Center for Electron Microscopy (ICEM), Indiana University School of Medicine, Indianapolis, IN USA; 4 Cambridge University Hospitals NHS Foundation Trust and the Cambridge Brain Bank, Cambridge, UK; 5 Department of Neurology, Erasmus Medical Centre, Rotterdam, The Netherlands; 6 Division of Neuropathology, National Hospital for Neurology and Neurosurgery, Queen Square, London, UK; 7 Department of Clinical and Movement Neurosciences, UCL Queen Square Institute of Neurology, London, UK; 8 Department of Cell and Developmental Biology, University College, London, UK; 9 Department of Pathology and Laboratory Medicine, Indiana University School of Medicine, Indianapolis, IN USA

**Correction: Acta Neuropathologica Communications (2024) 12:99**

10.1186/s40478-024-01813-z

Following publication of the original article [1], the sentence “It remains to be determined if the formation of TMEM106B filaments can influence the risk of developing neurodegenerative diseases” in the paragraph starting with “Abundant filaments made of residues” under Discussion heading gives the relevant meaning of the previous sentence. The author wants to delete the sentence.

The original article has been corrected.
