# Supplementary figures and images for: Cleaved TMEM106B forms amyloid aggregates in central and peripheral nervous systems
Source: Acta Neuropathol Commun. 2024 Jun 17;12:99. doi: 10.1186/s40478-024-01813-z (PMC11181561; doi:10.1186/s40478-024-01813-z)

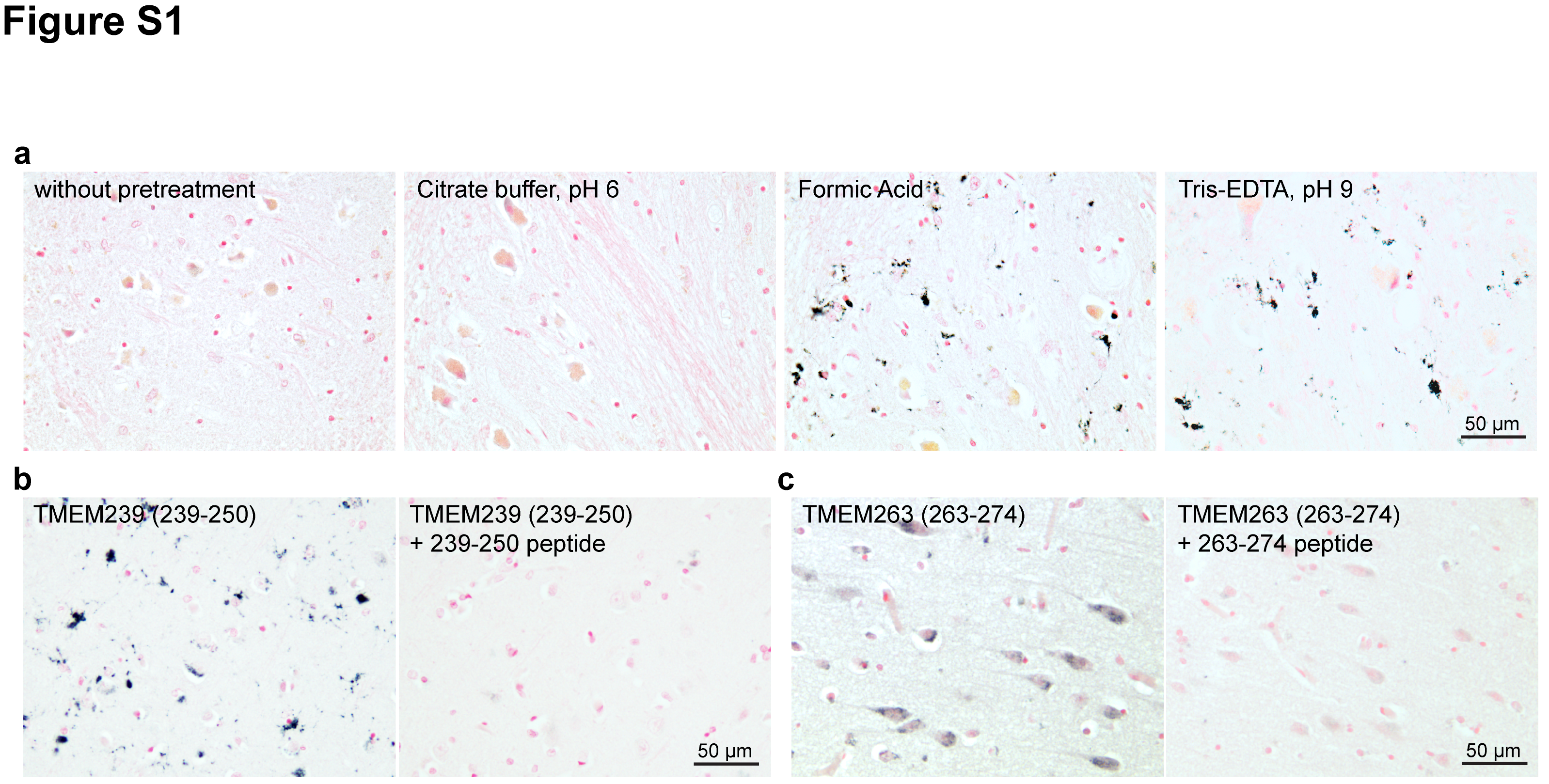

Supplement: Supplementary file 1 — Supplementary Figure 1. Immunohistochemical staining optimisation and specificity. (a), Several antigen retrieval methods were tested and provided different immunostaining results with TMEM239 antibody using medulla sections of a Parkinson’s disease case aged 73 years (case 39). No staining was observed after heat-induced epitope retrieval (HIER) with citrate buffer or without pretreatment, whereas formic acid treatment or HIER with Tris-EDTA buffer resulted in strong staining. Nuclei were counterstained in red. (b, c), Immunostaining with antibodies TMEM239 (b), and TMEM263 (c) of adjacent cortical sections from (b) a 78-year-old (case 3) and (c) a 73-year-old (case 70) individual with progressive supranuclear palsy. Following pre-adsorption of the antibodies with their respective immunogens, all the stainings were abolished. [file 40478_2024_1813_MOESM1_ESM.png]

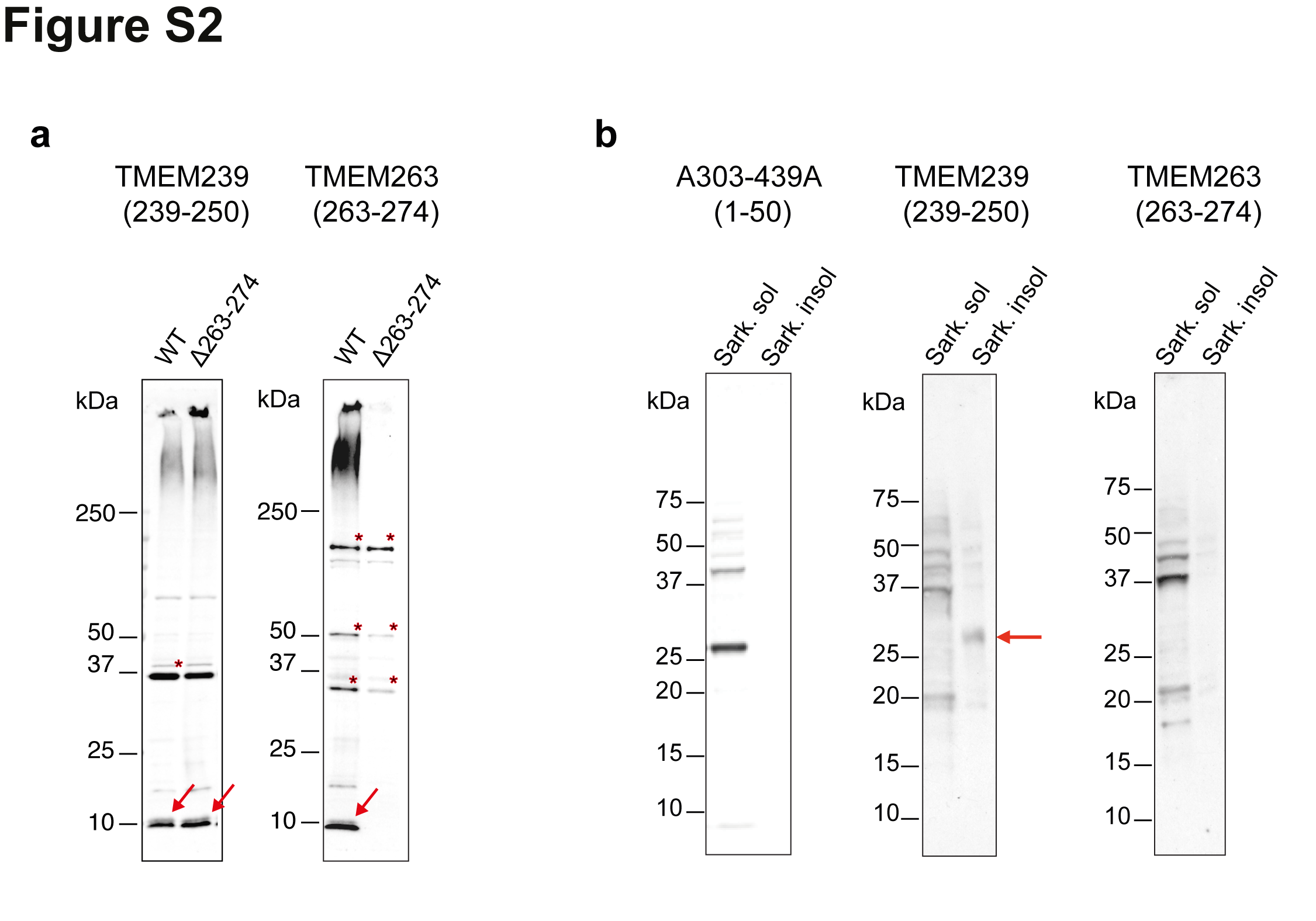

Supplement: Supplementary file 2 — Supplementary Figure 2. Characterisation of antibody TMEM263 by immunoblotting. (a), TMEM106B (120-274, WT) and TMEM106B (120-262, Δ263-274) were expressed in E. coli. The pellets from 1 ml bacterial cultures were used for immunoblotting with antibodies TMEM239 and TMEM263. Red arrows point to TMEM106B bands, whereas red asterisks indicate non-specific binding. Note that TMEM239 labelled both TMEM106B (120-274, WT) and TMEM106B (120-262, Δ263-274), whereas TMEM263 only labelled TMEM106B (120-274). (b), Immunoblots of sarkosyl soluble (Sark. sol) and sarkosyl-insoluble (Sark. insol) fractions from the frontal cortex of 58 yo neurologically normal individual (case 99) stained with antibodies A303-439A, TMEM239, and TMEM263. A 29kDa band is observed with the TMEM239 antibody in the sarkosyl-insoluble fraction, this band was not recognised by TMEM263, and A303-439A antibodies supporting the absence of the N-terminus and C-terminus of TMEM106B in the aggregates. The antibodies recognised similar bands in the sarkosyl-soluble fraction. [file 40478_2024_1813_MOESM2_ESM.png]

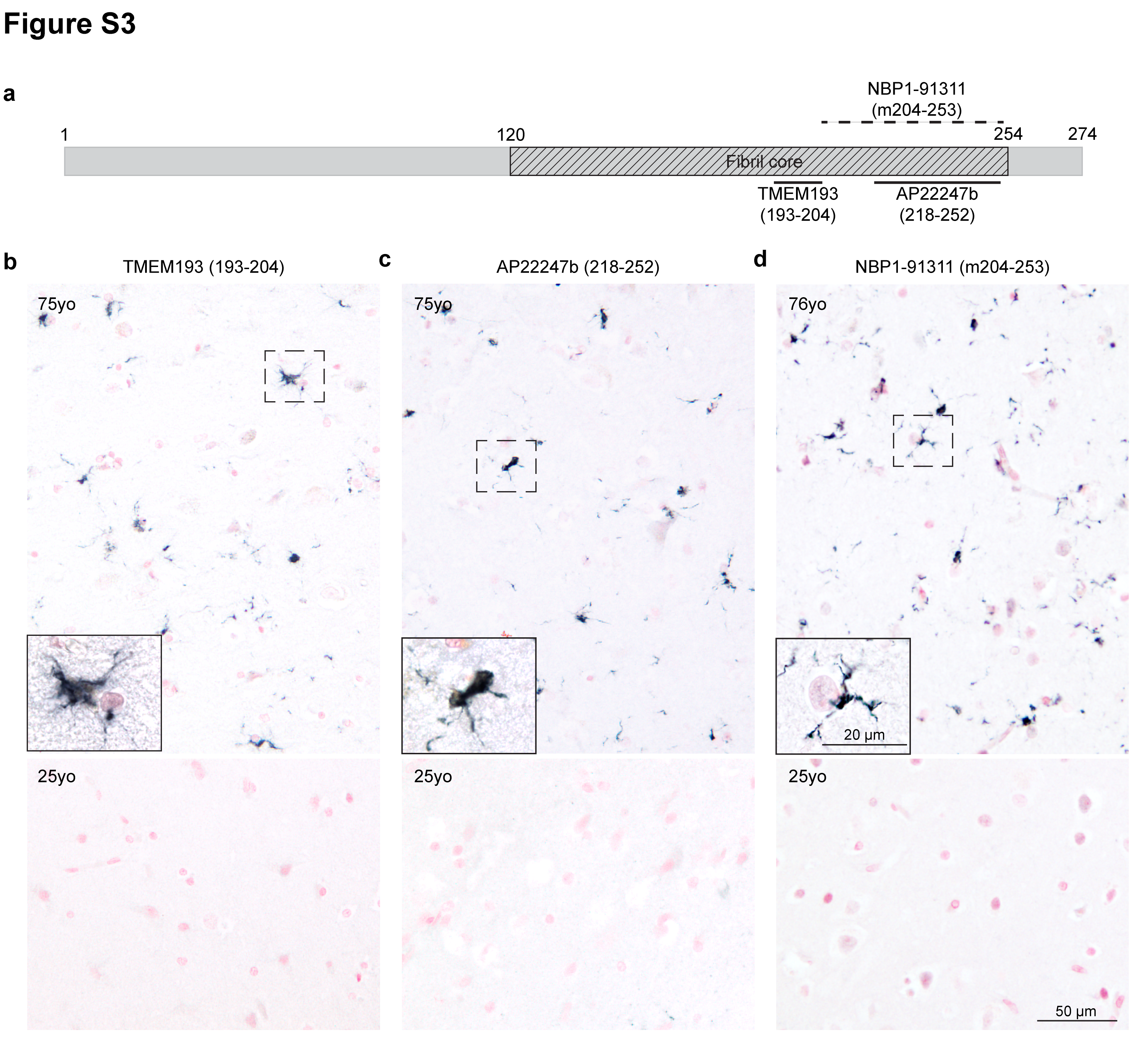

Supplement: Supplementary file 3 — Supplementary Figure 3. Staining of TMEM106B inclusions with antibodies TMEM193, AP22247b and NBP1-91311. (a), Schematic of human TMEM106B, with the epitopes of anti-TMEM106B antibodies indicated. (b), Staining of frontal cortex sections from neurologically normal controls, aged 25 (case 2), 75 (case 1) and 76 (case 6) (yo=years-old). Nuclei are counterstained in red. Scale bar, 50 µm and 20 µm (insets). Note the presence of TMEM106B inclusions in the brains of individuals aged 75 and 76 and the absence of staining in the brain from the 25-year-old individual. [file 40478_2024_1813_MOESM3_ESM.png]

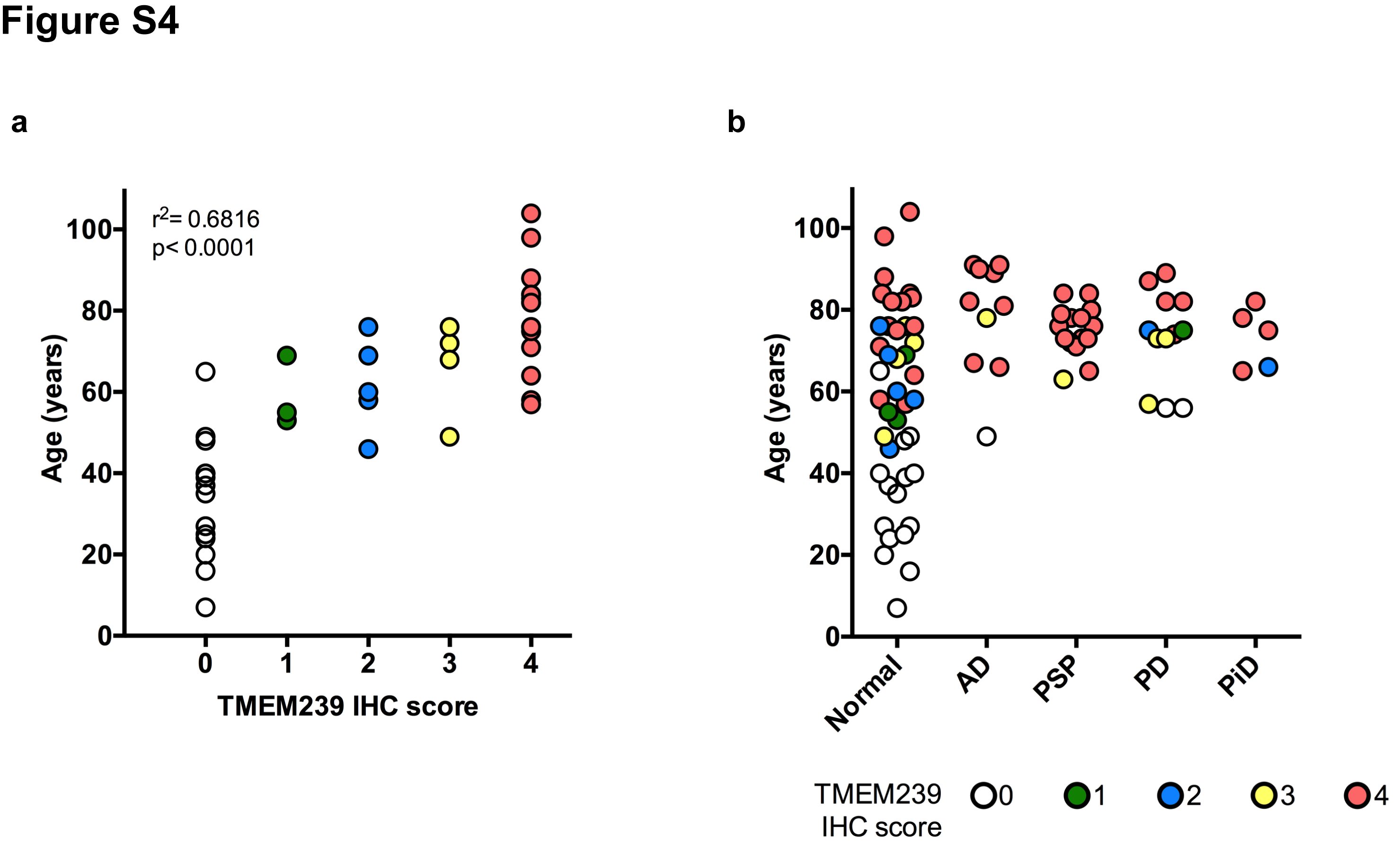

Supplement: Supplementary file 4 — Supplementary Figure 4. Presence of TMEM106B inclusions in relation to age in brain of healthy and diseased subjects. TMEM106B inclusion burden determined by TMEM239 IHC, and scored semiquantitavely by a researcher blinded to disease status and age. (a), TMEM239 IHC scores versus age in neurologically normal individuals and (b), in diseased individuals. Only disease cohorts which had at least 5 individuals analysed are indicated (b). In neurologically normal individuals as well as subjects affected by disease the amount of TMEM106B inclusions correlated with age. Each circle represents an individual in the cohort, and colour of the circles represents the TMEM239 IHC score. Semi-quantitative IHC score: 0 (white), 1 (green), 2 (blue), 3 (yellow), 4 (red). [file 40478_2024_1813_MOESM4_ESM.png]

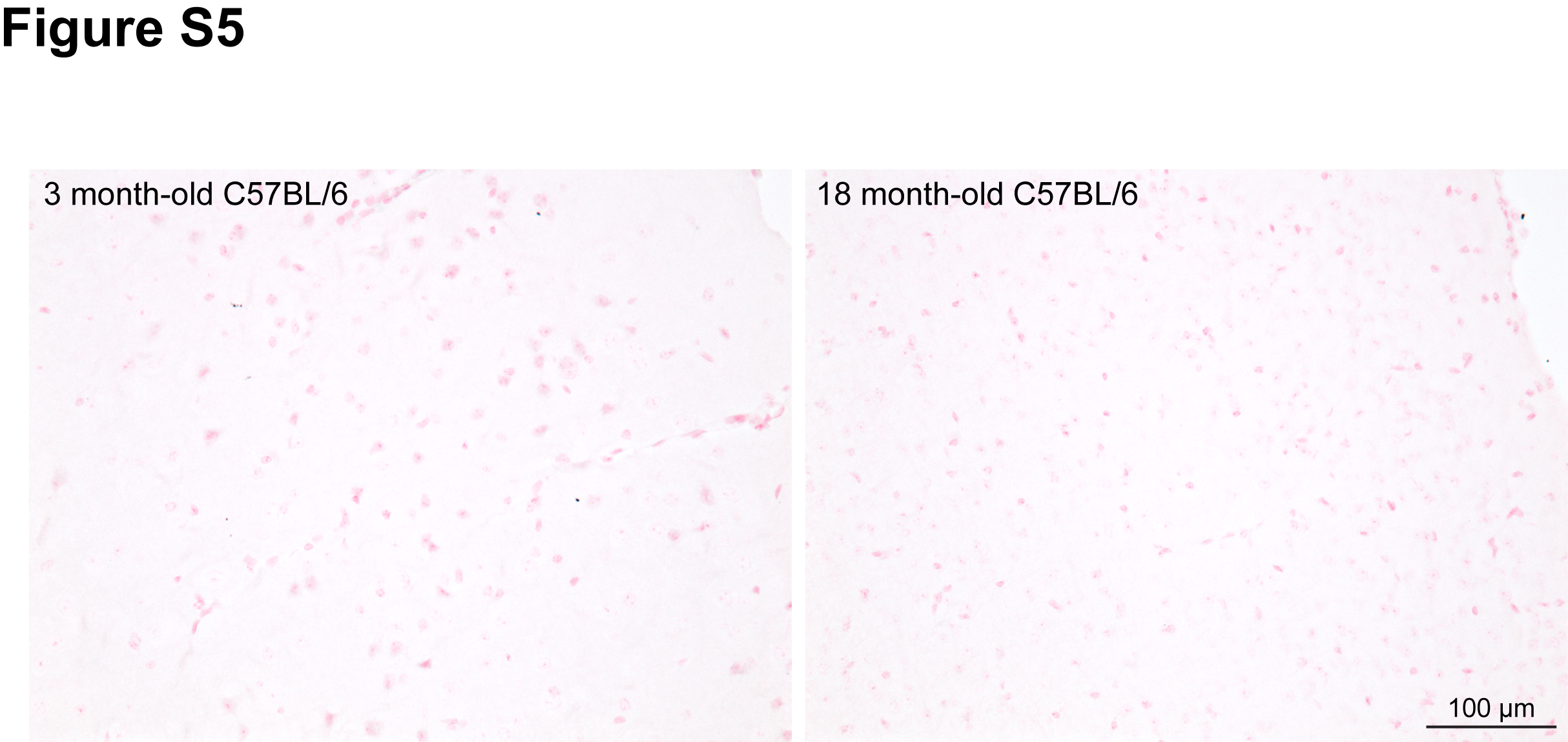

Supplement: Supplementary file 5 — Supplementary Figure 5. No TMEM106B inclusions were detected in the brains of young and aged mice. Staining with TMEM239 of representative cerebral cortex sections (treated with formic acid) from C57BL6/J mice aged 3 months and 18 months. Nuclei are counterstained in red. Scale bar, 100 µm. [file 40478_2024_1813_MOESM5_ESM.png]

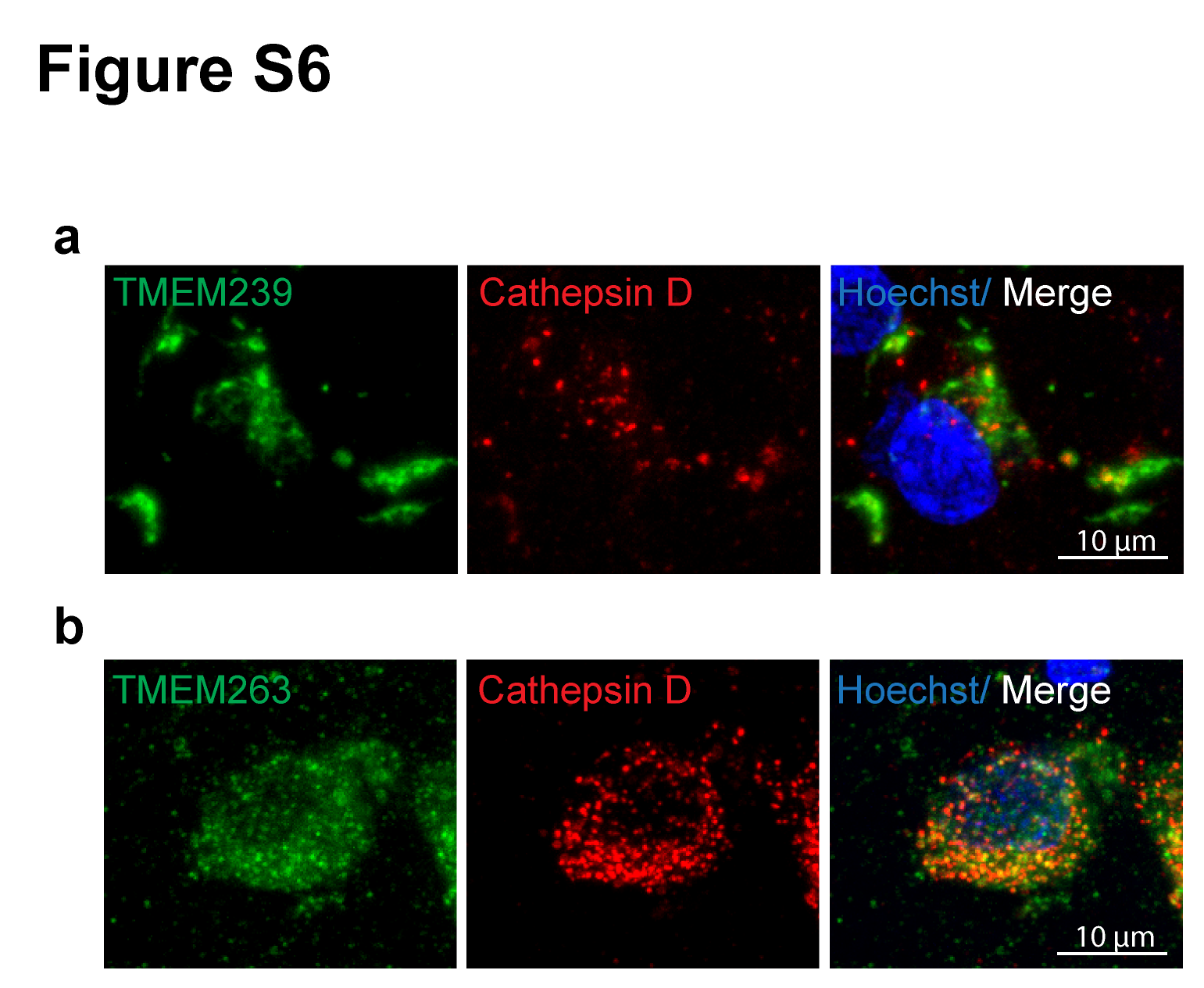

Supplement: Supplementary file 6 — Supplementary Figure 6. Double-labelling immunofluorescence of TMEM106B antibodies and a lysosomal marker. (a), Double-labelling immunofluorescence of sections from the frontal cortex of neurologically normal individuals aged 72 (case 107) with antibody TMEM239 (green) and lysosomal marker cathepsin D (red). Inclusions were labelled by TMEM239. Some TMEM106B inclusions co-localised with cathepsin D staining (double labelling in yellow) indicating their lysosomal localization. (b), Double-labelling immunofluorescence of sections from the frontal cortex of a neurologically normal individual aged 76 (case 108) with antibody TMEM263 (green) and lysosomal marker cathepsin D (red). TMEM263 staining co-localised with lysosomes (double labelling in yellow). [file 40478_2024_1813_MOESM6_ESM.png]
