# Supplementary material for: Cleaved TMEM106B forms amyloid aggregates in central and peripheral nervous systems
Source: Acta Neuropathol Commun. 2024 Jun 17;12:99. doi: 10.1186/s40478-024-01813-z (PMC11181561; doi:10.1186/s40478-024-01813-z)
Supplement: Supplementary file 7 — Supplementary Table 1. [file 40478_2024_1813_MOESM7_ESM.pdf]

Supplementary Table 1. Overview of cases (*Continued*)

Case	Disease	Age (yrs)	Gender	Mutation	Brain region; Peripheral organ	Cortical TMEM239 IHC score 0-4 (*)	References
**27**	Sanfilippo A syndrome	15	F	*SGSH*	FL	0	[45]
**28**	Neuroserpin	43	M	*SERPINI1*	DRG	0 (DRG)	[56]
**29**	Neuroserpin	39	M	*SERPINI1*	DRG	0 (DRG)	
**30**	Neuroserpin	41	F	*SERPINI1*	DRG	0 (DRG)	
**31**	MS	37	M	No	FL	0	
**32**	MS	59	F	No	MD	0 (MD)	
**33**	Motor Neuron Disease	64	M	*C9orf72*	FL, BS, SC	3	
**34**	LBD	67	M	No	Retina	0 (Retina)	
**35**	LBD	76	F	*GBA (E365K/E326K)*	FL	4	
**36**	PDD	74	M	*GBA (E365K/E326K)*	FL	4	
**37**	PD	56	F	*GBA (L444P)*	FL	0	
**38**	PD	56	M	*GBA (L444P)*	FL	0	
**39**	PD	73	F	No	Pons	-	
**40**	PD	73	M	No	FL, SN, HPC	3	
**41**	PD	75	M	No	FL, SN, HPC	1	
**42**	PD	75	F	No	FL, SN, HPC	2	
**43**	PD	82	M	No	FL, SN, HPC	4	
**44**	PD	89	M	No	FL, SN, HPC	4	
**45**	PD	87	F	No	FL, SN, HPC	4	
**46**	FAD	49	F	*APP (V717F)*	FL; Heart, Liver, Lung, Gut, Thyroid, Adrenal gland	0	[53]
**47**	FAD	67	F	*PSEN (F105)*	FL	4	[39]
**48**	AD	66	M	No	FL, BG	4	
**49**	AD	78	M	No	FL, BG	3	
**50**	AD	81	M	No	HPC	4	
**51**	AD	82	M	No	FL, BG	4	
**52**	AD	89	M	No	FL, BG	4	
**53**	AD	91	F	No	FL, BG	4	
**54**	VaD	94	M	No	FL	4	
**55**	Congophilic Angiopathy	71	M	No	FL, BS, Spinal cord	4	
**56**	AGD	84	M	No	HPC	4	[57]
**57**	FTDP-17T	54	F	*MAPT (+3)*	BS, Spinal cord	2 (Spinal cord)	[54, 55]
**58**	FTDP-17T	58	F	*MAPT (+3)*	Spinal cord	2 (Spinal cord)	[54, 55]
**59**	FTDP-17T	61	F	*MAPT (+3)*	Spinal cord	2 (Spinal cord)	[54, 55]
**60**	FTDP-17T	64	M	*MAPT (+3)*	BS, Spinal cord	2 (Spinal cord)	[54, 55]
**61**	PiD	53	F	*MAPT (ΔK281)*	DRG	0 (DRG)	

**62**	PiD	65	F	No	FL	4	
**63**	PiD	66	M	No	FL	2	
**64**	PiD	78	F	No	FL	4	
**65**	PiD	82	M	No	FL	4	
**66**	PSP	63	M	No	FL, BG	3	
**67**	PSP	65	F	No	FL, BG	4	
**68**	PSP	71	F	No	FL, BG	4	
**69**	PSP	72	M	No	FL, BG	4	
**70**	PSP	73	F	No	FL, BG	4	
**71**	PSP	73	M	No	FL, BG	4	
**72**	PSP	73	F	No	FL, BG	4	
**73**	PSP	76	M	No	FL, BG	4	
**74**	PSP	76	F	No	FL, OL	4	
**75**	PSP	79	F	No	FL, BG	4	
**76**	PSP	80	M	No	FL, BG	4	
**77**	PSP	84	F	No	FL, BG	4	
**78**	PSP	84	M	No	FL, BG	4	
**79**	AD + atypical tauopathy	91	M	No	FL, Spinal cord	4	
**80**	FTLD-TDP-C	74	M	No	FL, BG	4	
**81**	Dementia	48	M	No	FL, HPC	0	
**82**	Control	7	F	No	FL	0	[45]
**83**	Control	16	F	No	HPC	0	
**84**	Control	20	F	No	FL; Heart, Liver, Lymph nodes, Spleen	0	[39]
**85**	Control	24	F	No	FL	0	[39]
**86**	Control	27	F	No	FL, OL	0	
**87**	Control	27	M	No	FL, PS	0	
**88**	Control	35	M	No	FL, BG	0	
**89**	Control	37	F	No	FL; Heart, Liver, Lymph nodes, Spleen	0	[39]
**90**	Control	39	M	No	FL	0	[39]
**91**	Control	40	F	No	FL	0	[39]
**92**	Control	40	M	No	FL	0	[39]
**93**	Control	46	M	No	FL	2	[39]
**94**	Control	49	M	No	FL	3	[39]
**95**	Control	49	F	No	FL	0	[39]
**96**	Control	53	M	No	FL	1	[39]
**97**	Control	55	M	No	FL	1	[39]
**98**	Control	57	M	No	FL	4	[39]
**99**	Control	58	M	No	FL	2	[39]
**100**	Control	58	M	No	FL, SN, HPC	4	
**101**	Control	60	F	No	FL, BG	2	
**102**	Control	64	F	No	HPC	4	
**103**	Control	65	M	No	FL, BG	0	
**104**	Control	68	M	No	FL, BG	3	

**105**	Control	69	F	No	FL, BG	1	
**106**	Control	69	F	No	FL, BG	2	
**107**	Control	72	M	No	FL, BG	3	
**108**	Control	76	M	No	FL, BG	3	
**109**	Control	76	M	No	FL, BG	2	
**110**	Control	82	M	No	HPC, Pons	4	
**111**	Control	82	M	No	FL	4	[39]
**112**	Control	88	M	No	FL, SN, HPC	4	
**113**	Control	104	F	No	FL, SN, HPC	4	

AD: sporadic Alzheimer’s disease, AGD: argryophilic grain disease, CBD: corticobasal degeneration, Control: neurologically normal individual, FAD: familial Alzheimer’s disease, FTDP-17T: familial frontotemporal dementia parkinsonism linked to chromosome 17 caused by *MAPT* mutations, FTLD-TDP-C: sporadic frontotemporal lobar degeneration with TDP-43 inclusions type C, LBD: Lewy body dementia, MS: multiple sclerosis, PD: Parkinson’s disease, PDD: Parkinson ‘s disease dementia, PiD: Pick’s disease, PSP: Progressive supranuclear palsy, VaD: Vascular Dementia. FL: Frontal lobe, BG: Basal ganglia, BS: Brainstem, CBL: Cerebellum, HPC: Hippocampus, MD: Medulla, MB: Midbrain, OL: Occipital lobe, PL: Parietal lobe, TL: Temporal lobe, PS: Parasagittal, SC: Spinal cord, SN: Substantia nigra, Thal: Thalamus. Semiquantitative TMEM239 IHC score: 0 (none), 1 (mild), 2 (moderate), 3 (abundant), 4 (severe); *other brain regions than cortex

REFERENCES

[39] Schweighauser M, Arseni D, Bacioglu M, Huang M, Lövestam S, Shi Y et al (2022) Age-dependent formation of TMEM106B amyloid filaments in human brains. Nature 605:310-314.

[45] Winder-Rhodes SE, Garcia-Reitböck P, Ban M, Evans JR, Jacques TS, Kemppinen A et al (2012) Genetic and pathological links between Parkinson's disease and the lysosomal disorder Sanfilippo syndrome. Mov Disord 27:312-315.

[53] Roher AE, Kokjohn TA, Esh C, Weiss N, Childress J, Kalback W et al (2004) The human amyloid-beta precursor protein770 mutation V717F generates peptides longer than amyloid-beta-(40-42) and flocculent amyloid aggregates. J Biol Chem 279:5829-36.

[54] Spillantini MG, Goedert M, Crowther RA, Murrell JR, Farlow MR et al (1997) Familial multiple system tauopathy with presenile dementia: a disease with abundant neuronal and glial tau filaments. Proc Natl Acad Sci U S A 94:4113-4118.

[55] Spillantini MG, Murrell JR, Goedert M, Farlow MR, Klug A et al (1998) Mutation in the tau gene in familial multiple system tauopathy with presenile dementia. Proc Natl Acad Sci U S A 95:7737-7741.

[56] Takao M, Benson MD, Murrell JR, Yazaki M, Piccardo P et al (2000) Neuroserpin Mutation S52R Causes Neuroserpin Accumulation in Neurons and Is Associated with Progressive Myoclonus Epilepsy. J Neuropathol & Expl Neurol 59:1070-1086.

[57] Tolnay M, Spillantini M, Goedert M, Ulrich J, Langui D et al (1997) Argyrophilic grain disease: widespread hyperphosphorylation of tau protein in limbic neurons. Acta Neuropathol 93:477-484.
